# Exceptionally High Two‐Photon Absorption Cross Sections in Quinoidal Diazaacene‐Bithiophene Derivatives

**DOI:** 10.1002/anie.202503073

**Published:** 2025-04-10

**Authors:** Gabriel Sauter, Antonia Papapostolou, Audrey Pollien, Sergius Boschmann, Kathleen Fuchs, Pascal Merten, Kerstin Brödner, Frank Rominger, Jan Freudenberg, Uwe H. F. Bunz, Andreas Dreuw, Petra Tegeder

**Affiliations:** ^1^ Physikalisch‐Chemisches Institut Universität Heidelberg Im Neuenheimer Feld 253 69120 Heidelberg Germany; ^2^ Interdisziplinäres Zentrum für Wissenschaftliches Rechnen Universität Heidelberg Im Neuenheimer Feld 205A 69120 Heidelberg Germany; ^3^ Université Paris‐Saclay École Normale Supérieure Paris‐Saclay 4 Av. des Sciences Gif‐sur‐Yvette 91190 France; ^4^ Organisch‐Chemisches Institut Universität Heidelberg Im Neuenheimer Feld 270 69120 Heidelberg Germany

**Keywords:** Azaacenes, Heterophenoquinones, Nonlinear optics, Quantum chemical calculations, Two‐photon absorption

## Abstract

This study addresses the two‐photon absorption (2PA) properties of (azaacene‐annulated) heterophenoquinones through a synergistic approach combining detailed experimental and theoretical analyses. Exceptionally large 2PA cross sections are found over a broad spectral range in the near‐infrared spectral region, with values up to 4100 GM in the 1400–1600 nm range and even higher values of up to 51770 GM in the 850–950 nm range, which is outstanding for organic chromophores of this molecular size. Our quantum chemical calculations support the experimental findings and elucidate the underlying absorption mechanism leading to the corresponding 2PA properties. The occurrence of such large cross sections is explained by the high oscillator strength of the first excited singlet state and its strong coupling to higher excited electronic states. The large (state‐to‐state) transition dipole moments originate from the acceptor–π–donor–π–acceptor structure of the parent quinoidal bithiophene motif common to all compounds, which in addition also enables their optimal (anti)parallel alignment due to its symmetry and linearity.

## Graphical abstract

Unlocking new horizons in two‐photon absorption, this study uncovers how specially structured quinoidal‐bithiophene derivatives achieve remarkable two‐photon absorption cross sections in the near‐infrared range. Advanced calculations reveal the interplay of molecular symmetry and strong state coupling, shedding light on the mechanism behind their efficiency—potentially transforming fields like bioimaging, photodynamic therapy, and optical data storage.

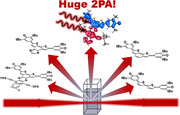

## Introduction

Two‐photon absorption (2PA) is one of the most basic and yet most important processes in the field of nonlinear optics, characterized by the simultaneous absorption of two photons by matter, which leads to an electronically excited state. This phenomenon stands in contrast to linear one‐photon absorption (1PA) and offers unique advantages. The nonlinear nature enables improved spatial selectivity, as it occurs only efficiently at the very focal point of a focused laser beam when a sufficiently high photon flux is reached. Furthermore, 2PA enables the use of longer excitation wavelengths with only half of the required energy, reaching even into the near‐infrared (NIR) spectral range, which allows for higher penetration depths into biological tissues without significant scattering or absorption. These aspects are particularly beneficial in biological application fields like high‐resolution microscopy and bioimaging,^[^ ^1^, ^2^, ^3^, ^4^ ^]^ as they enable detailed visualization of biological processes with minimal background noise and reduced tissue damage. Additionally, spatially precise delivery of energy opens up applications in various areas, such as nanofabrication processes,^[^ ^5^, ^6^ ^]^ targeted photodynamic therapy,^[^ ^7^ ^]^ and advanced optical data storage solutions,^[^ ^8^ ^]^ which are unattainable through conventional linear optical techniques. Most recently, strong nonlinear absorbers are also demanded in laser technologies, such as mode locking,^[^ ^9^ ^]^ pulse shaping, and stabilizing.^[^ ^10^ ^]^

**Figure 1:**
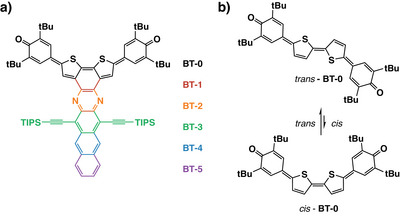
a) Investigated molecules and their nomenclature: starting from the parent quinoidal bithiophene (**BT‐0**) via **BT‐1** and **BT‐2** to the bithiophene‐based azaacenequinones with different acene lengths (**BT‐3**, **BT‐4**, and **BT‐5**). The numbering indicates the number of annulated rings. b) The *cis*‐*trans* isomerization of **BT‐0**.

In view of potential applications in photonics, great efforts have been made to synthesize materials with large two‐photon absorption cross sections (σ_2_) as well as to understand the relationship between molecular structure and nonlinear optical properties.^[^ ^11^ ^]^ Along this line of research, our study focuses here on the bithiophene‐based azaacenequinones (see Figure 1),^[^ ^12^ ^]^ whose isomers have recently been found to exhibit exceptionally strong nonlinear optical responses.^[^ ^13^ ^]^

To investigate the relationship between their 2PA properties and their molecular structures, we studied the influence of individual structural components in the azaacene derivatives, starting with the parent quinoidal bithiophene (**BT‐0**) followed by building up the largest azaacene (**BT‐5**) ring by ring (see Figure 1a). **BT‐0** belongs to a series of heteroquaterphenoquinones that have already demonstrated promising properties.^[^ ^14^ ^]^ In particular, the sulfur‐containing derivative exhibits an intense 1PA band in the NIR spectral region, suggesting its potential utility in high‐density optical storage materials for diode lasers and other applications. The π‐system of the azaacene moiety is perpendicular to the bithiophene moiety and can serve as an efficient electron acceptor, causing an additional push–pull effect that will also influence the electronic responsiveness of the chromophore. Our experimental investigations using the z‐scan method over a wide spectral range (680–980 nm and 1170–3000 nm) reveal that the 2PA cross sections of these molecular systems rank among the highest (up to 51770 GM) reported for organic compounds,^[^ ^15^, ^16^ ^]^ making them potential candidates for applications requiring high NIR‐2PA efficiencies. An interpretation of these enormous 2PA cross sections and a deep understanding of the 2PA mechanism are provided by calculations at the level of time‐dependent density functional theory (TDDFT) employing the so‐called sum‐over‐states (SOS) formalism.

## Results and Discussion

### Parent Quinoidal Bithiophene (**BT‐0**)

As shown by Takahashi et al.,^[^ ^14^ ^]^ the parent quinoidal bithiophene (**BT‐0**) exists in solution predominantly in the *trans* configuration, which is centrosymmetric (see Figure 1b). For centrosymmetric molecules, the selection rules governing one‐photon and two‐photon absorption are mutually exclusive. Thus, for symmetry reasons, an electronic transition that is visible in the linear 1PA spectrum is symmetry‐forbidden in the 2PA spectrum and vice versa.

**Figure 2:**
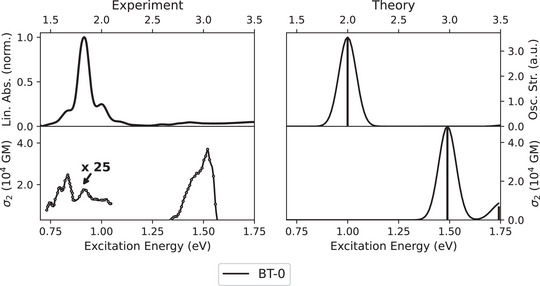
Comparison of the experimental 1PA and 2PA spectra of **BT‐0** in benzene with the corresponding spectra calculated at the CAM‐B3LYP‐D3(BJ)/aug‐cc‐pVDZ PCM(C_6_H_6_) level of theory. The 25x magnification applies only to the low‐energy band.

In the experimental UV–vis spectrum depicted in Figure 2, a distinct 1PA band is observed at 1.83eV (with shoulders at 1.66 and 2.00eV). This peak appears in the calculated spectrum with a slight blueshift of about 0.2eV and results from an excitation into the first excited singlet state (S_1_). The shoulders can be attributed to vibronic transitions, which are not present in the calculated spectrum since only electronic contributions were taken into account. Despite the centrosymmetry of *trans*‐**BT‐0**, this first excitation is not completely dark in the experimental 2PA spectrum, which may be due to several factors: the presence also of some *cis*‐**BT‐0** in the measured solution as well as vibrational distortions that break the strict selection rules. When examining the higher energy regime, the situation is exactly the opposite: there is no band in the linear absorption spectrum, but a peak with a very pronounced intensity is observed in the 2PA spectrum, which can be assigned to the second excited singlet state (S_2_). In the experiment, this transition appears at a photon energy of 1.53eV, which matches the calculated 2PA band at 1.49eV. This peak reaches a huge 2PA cross section of σ_2_ = 35550 GM.

**Figure 3:**
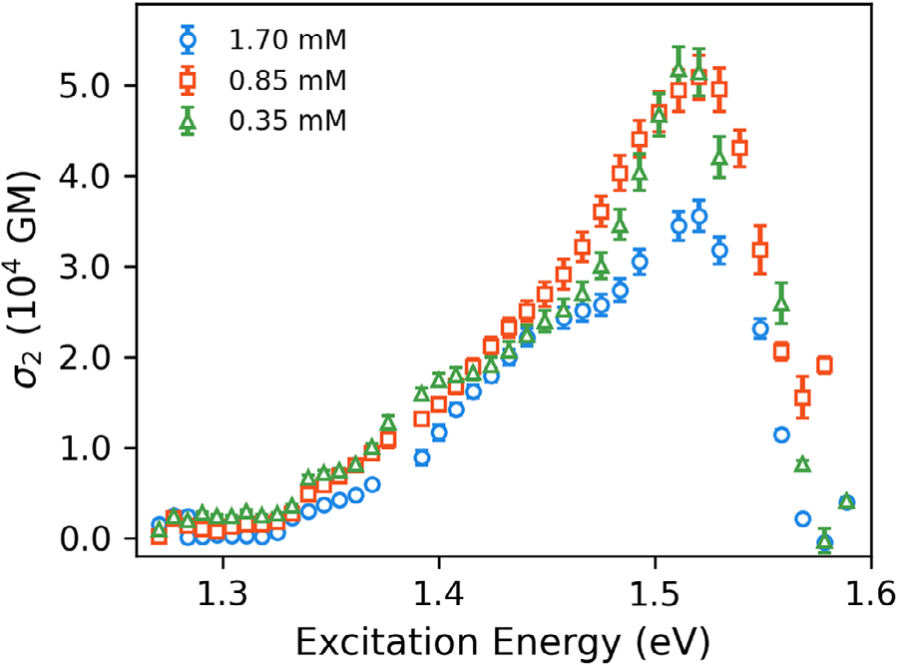
Concentration‐dependent 2PA spectra of **BT‐0** recorded in benzene.

Concentration‐dependent measurements (see Figure 3) reveal even higher cross sections of up to 51770 GM. This phenomenon can be explained by several factors. First, at lower concentrations, the influence of saturable absorption is reduced, allowing for a more accurate measurement of the true 2PA cross section. Additionally, as reported by Ajami et al.,^[^ ^17^ ^]^ the formation of molecular aggregates at higher concentrations can lead to a screening effect, where inner molecules are shielded from the incoming light, reducing the overall 2PA efficiency per molecule. As the concentration decreases, this screening effect is minimized, resulting in observing higher 2PA cross sections. Such giant 2PA cross sections are remarkable and unexpected for an organic molecule of this size and in the absence of incorporated metals.^[^ ^15^, ^16^ ^]^ Note that the 2PA spectra of all investigated molecular systems (see Figure 1) possess two distinct absorption bands at *E*_exc_ ≈ 0.8 − 0.9 eV (“low‐energy band”) and at *E*_exc_ > 1.3 eV (“high‐energy band”) (see Supporting Information Table S5).

Figure 2 shows that the calculations at the CAM‐B3LYP‐D3(BJ)/aug‐cc‐pVDZ PCM(C_6_H_6_) level of theory not only reproduce the experimental 1PA spectrum very well, but also the experimental 2PA spectrum. According to Beerepoot et al.,^[^ ^18^ ^]^ range‐separated functionals such as CAM‐B3LYP are well‐suited to correctly predict *qualitative trends* of 2PA strengths, even if the underestimation of excited‐state dipole moments as well as the overestimation of excitation energies leads to a tendency for the absolute values to be too low, albeit in a rather systematic way.

In order to gain deeper insight into the 2PA processes of **BT‐0** and thus explain the extraordinarily large 2PA cross section of the S_2_ state, the individual contributions of the SOS expression must be investigated. If two photons with the same energy ($\frac{\omega_{f}}{2}$) are absorbed, the so‐called SOS expression for a component of the 2PA transition moment between the ground state and the final state *f* reads

(1) $$S_{AB}^{0f}=\sum_{n\neq 0}\left(\frac{\left\langle 0|\mu_{A}|n\right\rangle \left\langle n|{\bar{\mu }}_{B}|f\right\rangle }{\omega_{n}-\frac{\omega_{f}}{2}}+\frac{\left\langle 0|\mu_{B}|n\right\rangle \left\langle n|{\bar{\mu }}_{A}|f\right\rangle }{\omega_{n}-\frac{\omega_{f}}{2}}\right)$$

where *A*, *B* ∈ *x*, *y*, and *z* denote the Cartesian components of the electric dipole operator $\vec{\mu }$. As can be seen from Equation (1), several properties contribute to the 2PA cross sections: the transition dipole moments between ground state and excited states ($\left\langle 0|\vec{\mu }|n\right\rangle$), the transition dipole moments between the final state and other excited states (for *n* ≠ *f*: $\left\langle n|\vec{\mu }|f\right\rangle$), and the difference dipole moment between the ground state and the final state (for *n* = *f*: $\left\langle f|\vec{\bar{\mu }}|f\right\rangle$) as well as the difference between the excitation energies and the energy of the incident photons ($\omega_{n}-\frac{\omega_{f}}{2}$). The calculated molecular properties relevant for this study are summarized in Table 1.

**Table 1:** Excitation energies (ω_*f*_), oscillator strengths, 2PA cross sections (σ_2_), transition dipole moments from the ground state ($\left\langle 0|\vec{\mu }|f\right\rangle$), difference dipole moments ($\left\langle f|\vec{\bar{\mu }}|f\right\rangle$), and transition dipole moments from the first excited state to the final state ($\left\langle 1|\vec{\mu }|f\right\rangle$) for the first five excited singlet states of **BT‐0** calculated at the CAM‐B3LYP‐D3(BJ)/aug‐cc‐pVDZ PCM(C_6_H_6_) level of theory.

*f*	ω_*f*_ (eV)	osc. str. (a.u.)	σ_2_ (GM)	$\left|\left\langle 0|\vec{\mu }|f\right\rangle \right|$ (a.u.)	$\left|\left\langle f|\vec{\bar{\mu }}|f\right\rangle \right|$ (a.u.)	$\left|\left\langle 1|\vec{\mu }|f\right\rangle \right|$ (a.u.)
1	2.00	3.550	0	8.513	0.001	−
2	2.98	0.000	48292	0.000	0.001	7.811
3	3.12	0.000	0	0.002	0.202	0.011
4	3.12	0.000	0	0.002	0.202	0.012
5	3.48	0.000	7002	0.001	0.006	1.317

**Figure 4:**
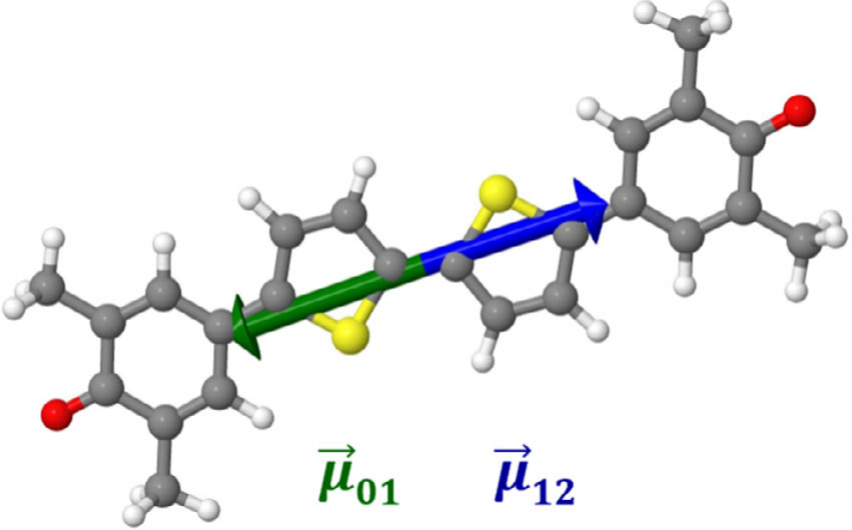
Visualization of $\left\langle 0|\vec{\mu }|1\right\rangle$ (green) and $\left\langle 1|\vec{\mu }|2\right\rangle$ (blue) transition dipole moment vectors in **BT‐0** calculated at the CAM‐B3LYP‐D3(BJ)/aug‐cc‐pVDZ PCM(C_6_H_6_) level of theory.

Resonance effects can be ruled out in the energy range under consideration, which is why the excitation energy ω_*f*_ is of rather minor importance in this analysis. However, since we only look at 2PA processes in which the final state does not yet have twice the energy of S_1_, it can generally be said that the SOS contribution decreases as ω_*n*_ increases. Due to the centrosymmetry, the states with the large σ_2_ themselves have an oscillator strength close to zero and also possess no dipole moments, which is why the terms for *n* = *f* in the sum in Equation (1) cannot make any contribution. However, the particularly bright S_1_ state of **BT‐0** in the UV–vis spectrum (see Figure 2) is striking. Consequently, those excited states that have a high transition dipole moment with the S_1_ state should also have a high 2PA transition moment, since the term for *n* = 1 then has a particularly large contribution in the sum in Equation (1), which is confirmed by our calculations (see Table 1). As is usual with centrosymmetric chromophores, the virtual state in the 2PA process can therefore be adequately described by the superposition of the ground and final states with only one other excited state (namely, S_1_, see Figure S30).^[^ ^11^ ^]^ The use of a corresponding three‐state model reveals that the 2PA cross section is particularly large if $\left\langle 0|\vec{\mu }|1\right\rangle$ and $\left\langle 1|\vec{\mu }|f\right\rangle$ are aligned either parallel or antiparallel to each other,^[^ ^19^ ^]^ which is the case here for the S_2_ state (see Figure 4).

**Figure 5:**
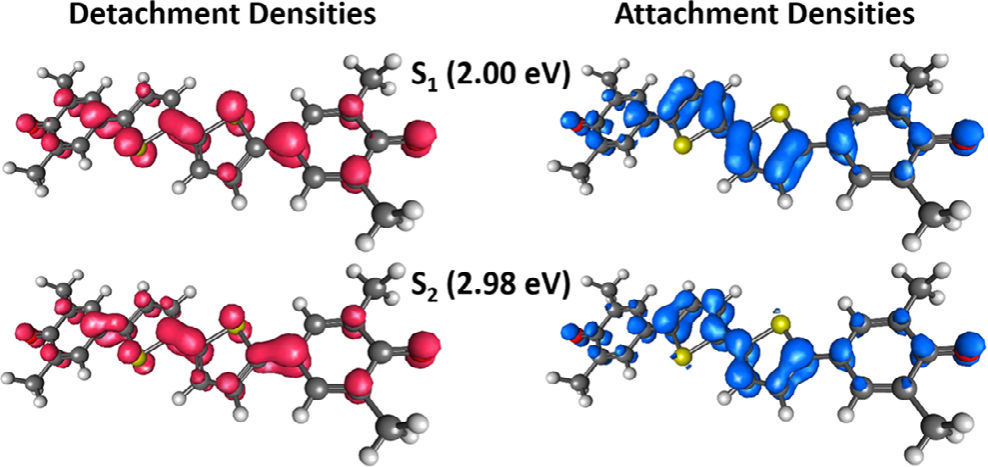
Attachment (blue) and detachment (red) densities of the first two excited singlet states of **BT‐0** calculated at the CAM‐B3LYP‐D3(BJ)/aug‐cc‐pVDZ PCM(C_6_H_6_) level of theory. The other excited states can be found in Figure S29.

**Figure 6:**
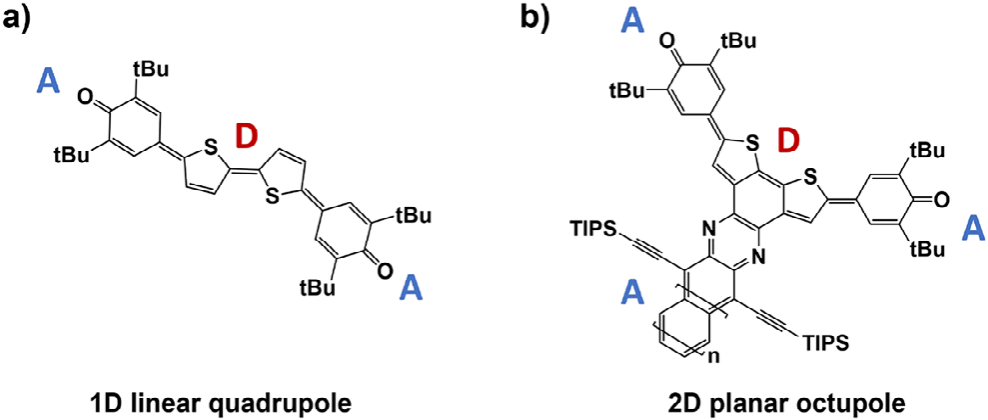
a) **BT‐0** offers a linear quadrupolar A–π–D–π–A structure. b) The azaacene moiety serves as an additional electron acceptor, resulting in a planar 2D octupolar structure with a small dipole moment in the direction of the acene.

The high σ_2_ of the S_2_ state thus results from the strongly allowed one‐photon transition into the S_1_ state in combination with the strong coupling of the first two excited singlet states with each other. Looking at the attachment and detachment densities of these two states in Figure 5, their great similarity is also noticeable. The detachment density corresponds to that part of the electron density that is replaced by the attachment density within the excitation process.^[^ ^20^, ^21^ ^]^ The high transition dipole moments originate from the quadrupolar structure of **BT‐0** comprising a central donor unit flanked by π‐conjugated linkers and terminal acceptor groups (A–π–D–π–A), as illustrated in Figure 6a.^[^ ^22^ ^]^ It has already been demonstrated that conjugated molecules, when substituted with donor or acceptor groups in an essentially centrosymmetric pattern, exhibit significantly enhanced 2PA cross sections, often surpassing the corresponding unsubstituted molecules by at least an order of magnitude.^[^ ^23^, ^24^, ^25^, ^26^ ^]^ In addition, the linearity of the structure enables the perfect alignment of the transition dipole moments.

### Loss of Centrosymmetry (**BT‐1**) and Annulation of the Pyrazine (**BT‐2**)

Benzannulation of **BT‐0** leads to rigid, C_2v_‐symmetric **BT‐1** (see Figure 1), a compound in which the *trans*‐configuration has been eliminated. Further pyrazannulation to **BT‐2** (Figure 1 and see SI, Section 2, for synthetic details concerning **BT‐2**) introduces an additional push–pull effect from the electron‐donating sulfur sites to the newly added electron‐accepting 1,4‐diazine unit resulting in an overall octupolar structure (see Figure 6b).^[^ ^22^ ^]^

**Figure 7:**
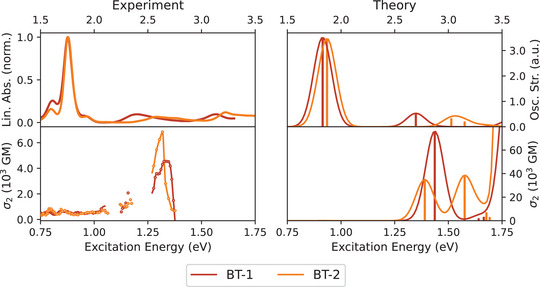
Comparison of the experimental 1PA and 2PA spectra of **BT‐1** and **BT‐2** in benzene with the corresponding spectra calculated at the CAM‐B3LYP‐D3(BJ)/aug‐cc‐pVDZ PCM(C_6_H_6_) level of theory.

Both the 1PA and 2PA spectra of **BT‐1** and **BT‐2** (see Figure 7) are very similar to those of **BT‐0**, the peaks are essentially only slightly red‐shifted. Although the molecules no longer possess an inversion center, the S_1_ states, which still exhibit the largest one‐photon transition strengths, have only small 2PA cross sections and also the calculated σ_2_ are almost negligible. In contrast, the high‐energy bands at 1.34 and 1.32eV in the experimental 2PA spectra of **BT‐1** and **BT‐2**, respectively, are again significantly stronger. However, the calculated 2PA peaks are much more pronounced than the measured ones, indicating experimental limitations in extracting the full σ_2_ due to the competing linear absorption that already begins in this region as a result of vibrational broadening. Although these are no longer centrosymmetric chromophores, the S_1_ state still makes the only significant contribution in the SOS expression in Equation (1) (see Figures S31 and S32). Thus, there is still a correlation between a high 2PA cross section and a large transition dipole moment between the S_1_ state and the final state of the 2PA process (see Table S6).

### Extension to the Azaacenes (**BT‐3**, **BT‐4**, and **BT‐5**)

**Figure 8:**
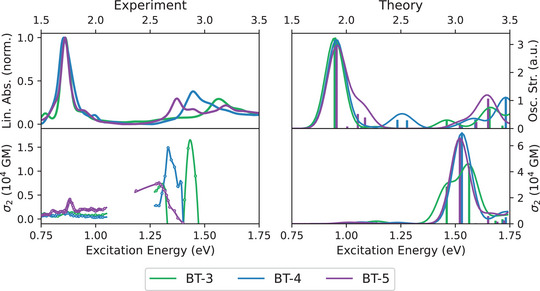
Comparison of the experimental 1PA and 2PA spectra of **BT‐3**, **BT‐4**, and **BT‐5** in benzene with the corresponding spectra calculated at the CAM‐B3LYP‐D3(BJ)/aug‐cc‐pVDZ PCM(C_6_H_6_) level of theory.

When the diazine is extended by further annulated phenyl rings (see SI, Section 2, for synthetic details concerning **BT‐3** and **BT‐4**), a strong 2PA can still be observed for the high‐energy band, which occurs at excitation energies of 1.30eV and above (see Figure 8).

**Figure 9:**
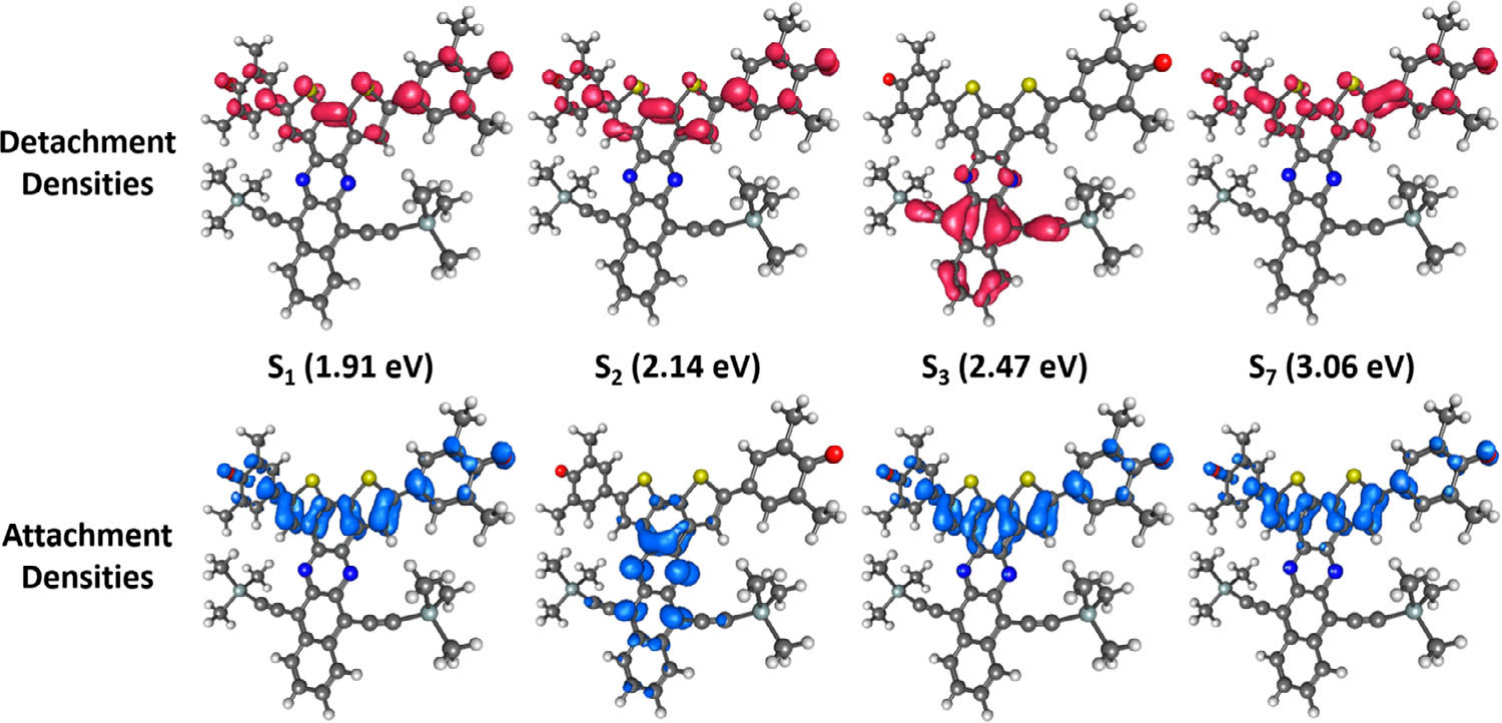
Attachment (blue) and detachment (red) densities of selected excited states of **BT‐4** calculated at the CAM‐B3LYP‐D3(BJ)/aug‐cc‐pVDZ PCM(C_6_H_6_) level of theory. Only the S_1_ state and other excited singlet states with significant σ_2_ are shown.

**Figure 10:**
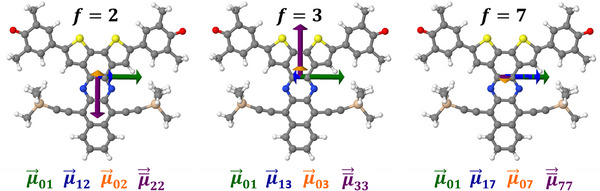
Visualization of $\left\langle 0|\vec{\mu }|1\right\rangle$ (green), $\left\langle 1|\vec{\mu }|f\right\rangle$ (blue), and $\left\langle 0|\vec{\mu }|f\right\rangle$ (orange) transition dipole moment vectors and $\left\langle f|\vec{\bar{\mu }}|f\right\rangle$ (purple) difference dipole moment vectors in **BT‐4** calculated at the CAM‐B3LYP‐D3(BJ)/aug‐cc‐pVDZ PCM(C_6_H_6_) level of theory.

For **BT‐3**, there is a double peak structure with σ_2_(*E*_exc_ = 1.31 eV) = 7250 GM and $\sigma_{2}\left(E_{\text{exc}}=1.43\;\mathrm{eV}\right)=14800\;\text{GM}$, which can also be seen in the calculated spectrum. As the number of annulated rings increases, the magnitude of σ_2_ remains unchanged in the high‐energy region, whereas the 2PA becomes stronger in the low‐energy regime. For a detailed analysis only the data of **BT‐4** will be discussed here, the corresponding data for the two other compounds can be found in the Supporting Information. The observation of the high‐energy bands to remain almost unchanged when comparing the calculated spectra (Figure 8) with the data of **BT‐0** (Figure 2) can be explained by the localization of the corresponding excitations at the bithiophene unit. The acene has only a minor influence, as can be seen from the attachment and detachment densities of the S_1_ and S_7_ states (see Figure 9). Consequently, the S_1_ and S_7_ states in **BT‐4** correspond to the S_1_ and S_2_ states in **BT‐0**, respectively. Compared to **BT‐0**, however, there are additional low‐lying excitations that also involve the acene moiety. The TDDFT calculations show that two new states emerge that are visible in both the 1PA and 2PA spectra (S_2_ state at 2.14eV and S_3_ state at 2.47eV). In both cases, these involve charge‐transfer excitations that extend over the entire molecule: for the S_2_ state from the bithiophene unit to the acene unit and for the S_3_ state vice versa (see Figure 9). The enhanced absorption bands compared to **BT‐0** between 0.75 and 1.00eV in the experimental 2PA spectra (see Figure 8) are therefore not due to excitations into the S_1_ states, but to these charge‐transfer excitations, which, depending on the molecule, are only about 0.1–0.3eV higher in energy than S_1_ and have a significantly larger 2PA cross section (see Table S7). A slight shift in peak position between experimental 1PA and 2PA spectra is common due to vibronic effects not accounted for in our calculations.^[^ ^27^ ^]^ In contrast to the excitations that are localized only at the bithiophene unit and are not affected by the acene (i.e., S_1_ and S_7_ for **BT‐4**), the excitations with charge‐transfer character (i.e., S_2_ and S_3_ for **BT‐4**) are increasingly red‐shifted with increasing acene length (see Table S7). In the case of **BT‐5**, a σ_2_ of up to 4200 GM can be measured as a result of these charge‐transfer excitations at 0.88eV. The calculated values are smaller, but a severe underestimation of absolute 2PA transition strengths by range‐separated functionals is already known and the trends are correctly reproduced.^[^ ^18^, ^28^ ^]^ It can also be observed that the 2PA cross section still correlates with the transition strength between the S_1_ state and the final state (see Table S7). For all states with significant σ_2_ (S_2_, S_3_, and S_7_ in **BT‐4**), $\left\langle 0|\vec{\mu }|1\right\rangle$ and $\left\langle 1|\vec{\mu }|f\right\rangle$ are aligned either parallel or antiparallel to each other (see Figure 10). However, since the quinoidal azaacenes have no inversion center, the oscillator strengths of the final states in the 2PA processes are no longer zero. Furthermore, in particular, for the excitations with charge‐transfer character the change in dipole moment is significant and becomes even larger with increasing acene length, which is why the terms for *n* = *f* in the sum in Equation (1) also make a contribution (see Figures S34 and S35). When considering a corresponding four‐state model, it becomes clear that for the S_2_ and S_3_ states the four (transition) dipole moment vectors are not optimally aligned, since $\left\langle 0|\vec{\mu }|1\right\rangle$ and $\left\langle 1|\vec{\mu }|f\right\rangle$ as well as $\left\langle 0|\vec{\mu }|f\right\rangle$ and $\left\langle f|\vec{\bar{\mu }}|f\right\rangle$ should each have the same orientation, i.e., either parallel or antiparallel (see Figure 10).^[^ ^19^ ^]^

## Conclusion

In summary, our comprehensive study has revealed exceptional 2PA properties of (azaacene‐annulated) heterophenoquinones and elucidated the underlying mechanism through the combined use of experimental and quantum chemical methods. The largest 2PA cross sections, which reach measured values of up to 51770 GM in a region between 850 and 950 nm, can be traced back to excitations localized at the parent quinoidal bithiophene molecular framework. This can be explained by the very strongly 1PA‐allowed S_1_ state and the linear as well as symmetric A–π–D–π–A structure, which favors high state‐to‐state transition dipole moments with optimal (anti)parallel alignment. The introduction of an additional azaacene unit leads to further excitations (with charge transfer character between the bithiophene and acene units), which also exhibit large 2PA cross sections of up to 4200 GM in a region between 1400 and 1600 nm. This capability for multiple 2PA processes with exceptionally large cross sections across different wavelength ranges within a single chromophore highlights the potential of these materials for advanced NIR‐2PA applications.

## Supporting Information

The authors have cited additional references in the Supporting Information.^[^ ^28^, ^29^, ^30^, ^31^, ^32^, ^33^, ^34^, ^35^, ^36^, ^37^, ^38^, ^39^, ^40^, ^41^, ^42^, ^43^, ^44^, ^45^, ^46^, ^47^ ^]^

## Conflict of Interests

The authors declare no conflict of interest.

## Supporting information

Supporting Information

Supporting Information

## Data Availability

Deposition Numbers CCDC 2411868 (**S7**), CCDC 2411869 (**BT‐3**), and CCDC 2411870 (**BT‐4**) contain the supplementary crystallographic data for this paper. These data are provided free of charge by the joint Cambridge Crystallographic Data Centre and Fachinformationszentrum Karlsruhe Access Structures service.
