# Supplementary material for: Exceptionally High Two‐Photon Absorption Cross Sections in Quinoidal Diazaacene‐Bithiophene Derivatives
Source: Angew Chem Int Ed Engl. 2025 Apr 10;64(23):e202503073. doi: 10.1002/anie.202503073 (PMC12124434; doi:10.1002/anie.202503073)
Supplement: Supplementary file 1 — Supporting Information [file ANIE-64-e202503073-s001.pdf]

Supporting Information: Exceptionally High Two-Photon Absorption Cross Sections in Quinoidal Diazaacene-Bithiophene Derivatives

Gabriel Sauter,+,[a] Antonia Papapostolou,+,[b] Audrey Pollien,[a,c] Sergius Boschmann,[a] Kathleen Fuchs,[d] Pascal Merten,[d] Kerstin Brödner,[d] Frank Rominger,[d] Jan Freudenberg,[d] Uwe H. F. Bunz,[d] Andreas Dreuw,*[b] and Petra Tegeder*[a]

[a] Gabriel Sauter, Audrey Pollien, Sergius Boschmann, Petra Tegeder
Physikalisch-Chemisches Institut, Universität Heidelberg, Im Neuenheimer Feld 253, 69120 Heidelberg, Germany.
E-mail: tegeder@uni-heidelberg.de
[b] Antonia Papapostolou, Andreas Dreuw
Interdisziplinäres Zentrum für Wissenschaftliches Rechnen, Universität Heidelberg, Im Neuenheimer Feld 205A, 69120 Heidelberg, Germany.
E-mail: dreuw@uni-heidelberg.de
[c] Audrey Pollien
Université Paris-Saclay, École Normale Supérieure Paris-Saclay, 4 Av. des Sciences, 91190 Gif-sur-Yvette, France.
[d] Kathleen Fuchs, Kerstin Brödner, Jan Freudenberg, Frank Rominger, Uwe H. F. Bunz
Organisch-Chemisches Institut, Universität Heidelberg, Im Neuenheimer Feld 270, 69120 Heidelberg, Germany.
[+] These authors contributed equally to this work.

Table of Contents

Contents

1. General Remarks 3

2. Synthetic Procedures 4

3. VT-NMR 13

4. NMR Spectroscopy 18

5. Crystallographic Data 26

6. Supplementary Spectroscopic Data 29

7. Experimental Methods 30

7.1. Two-Photon Absorption Measurements . . . 30

7.1.1. Sample Preparation . . . 30

7.1.2. Open-Aperture Z-Scan Technique . . . 30

7.1.3. Analysis . . . 30

7.1.4. Saturable Absorption . . . 31

8. Computational Methodology 31

8.1. Computational Details . . . 31

8.2. Analysis . . . 31

9. Experimentally determined 1PA and and 2PA properties of all investigated compounds 32

9.1. Concentration-Dependent 2PA Measurements . . . 32

9.2. Reversed Saturable Absorption . . . 33

10. Calculated Excited-State Properties and Few-State Models 34

1. General Remarks

Reagents and Solvents for Synthesis

All reagents and solvents were obtained from commercial suppliers (SIGMA-ALDRICH, TCI Deutschland GmbH, ALFA AESER, FISHER SCIENTIFIC, VWR, and ABCR GmbH) and used without further purification. Deuterated solvents for NMR analysis were purchased from MERCK. Absolute solvents were used directly dispensed from a solvent system (MB SPS-800) containing sufficient drying agents.

Flash Column Chromatography

Flash column chromatography was carried out using silica gel (grain size 0.04 - 0.063 mm) produced by SIGMA-ALDRICH. As the mobile phase, the solvents named in the synthetic procedure were used. For thin-layer chromatography, Polygram Sil g/UV 254 plates from Macherey Nagel were used and examined under UV light irradiation (254 nm and 365 nm).

Nuclear Magnetic Resonance Spectroscopy

All NMR spectra were recorded in deuterated solvents ($CDCl_3$ and CD_2Cl_2) at room temperature (if not stated otherwise) on a BRUKER Avance III (400 MHz), BRUKER Avance III (600 MHz) or BRUKER Avance III (700 MHz). ^{13}C NMR spectra were measured proton decoupled if not stated otherwise. Chemical shifts δ are reported in parts per million (ppm) and coupling constants J in Hz. All spectra were referenced to the solvent signal.[1] To describe multiplicities, the following abbreviations are used: s = singlet, d = doublet, t = triplet, m = multiplet. The spectra were processed and integrated using the MestreNova processor.

Mass Spectrometry

High-resolution mass spectra (HRMS) were obtained by matrix-assisted laser desorption ionization (MALDI) using DCTB as matrix or direct analysis in real-time (DART) experiments on a BRUKER ApexQe hybrid 9.4 T FT-ICR or a BRUKER AutoFlex Speed time-of-flight spectrometer.

IR Spectroscopy

IR spectra were recorded on a JASCO FT/IR-4100 using the neat compounds at room temperature. The data were processed using JASCO Spectra ManagerTM II. All signals are reported in wavenumbers [cm^{-1}].

Melting Points

Melting points were determined in open glass capillaries with a melting point apparatus MEL-TEMP, Electrothermal, Rochford, UK.

X-ray Single-Crystal Structure Analysis

X-ray single-crystal structure analyses were measured on a BRUKER Smart APEX-II QuazarArea detector or STOE Stadivari CCD area detector diffractometer. Diffraction intensities were corrected for Lorentz and polarization effects. An empirical absorption correction was applied using SADABS[2] based on the Laue symmetry of reciprocal space or using X-Area LANA 1.70.0.0 (STOE, 2017) based on the Laue symmetry of the reciprocal space. Hydrogen atoms were either isotropically refined or calculated. The structures were solved and refined using the SHELXT-2014[3] and refined against F^2 with a Full-matrix least-squares algorithm using the SHELXL-2018/3 software.[3]

2. Synthetic Procedures

2,4,6-Tris[3,5-bis(1,1-dimethylethyl)-4[(trimethylsilyl)oxy]-phenyl]boroxine (**S1**)[4,5], dibromide precursors **S2**[6], **S4**[7], **S6**[8] and **S8**[8] as well as diradical **BT-5**[9] were synthesized according to literature procedures.

General procedure for Suzuki coupling reactions (GP1):

To a degassed mixture of THF/water (10:1, v:v) was added the corresponding dibromide precursor (1.00 equiv.), **S1** (1.00 equiv.), $Pd(PPh_3)_4$ (0.10 equiv.) and K_2CO_3 (9.00 equiv.) under argon atmosphere. The mixture was heated to 95 °C overnight and extracted with DCM. The combined organic layers were washed with water and dried over $MgSO_4$. The crude product was purified by flash column chromatography (silica gel, PE/DCM or PE/EE).

Note: Synthesized 2,4,6-tris[3,5-bis(1,1-dimethylethyl)-4-[(trimethylsilyl)oxy]phenyl]boroxine (**S1**) used for Suzuki coupling contained 10% of its free boronic acid (as observed in literature).[10]

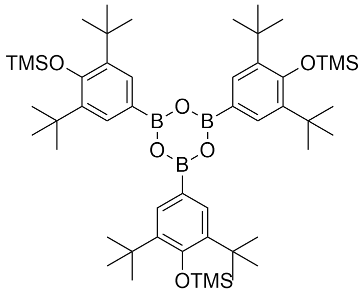

S1

General procedure for oxidation reaction (GP2):

To a 1:1 mixture of dry THF and aqueous KOH (0.1 M) was added the corresponding bisphenol (1.00 equiv) and $K_3[Fe(CN)_6]$ (2.00 equiv.) under argon atmosphere at room temperature. The mixture was stirred for 15 min and turned dark immediately. The reaction mixture was extracted with DCM, washed with water, dried over $MgSO_4$ and dried *in vacuo*.

4,4'-(Benzo[2,1-*b*:3,4-*b*']dithiophene-2,7-diyl)bis(2,6-di-*tert*-butylphenol) (S3)

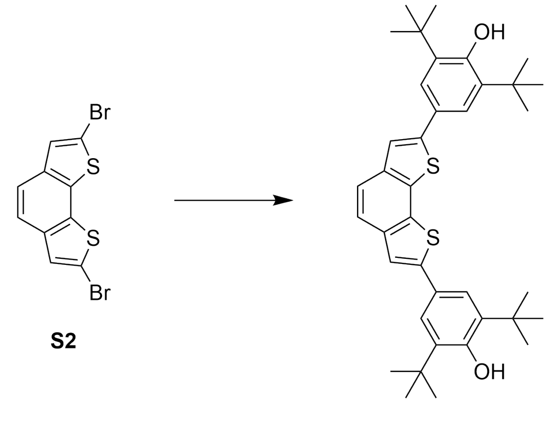

S2

S3

GP1 was applied to **S2** (391 mg, 1.12 mmol, 1.00 equiv.), **S1** (1.13 g, 1.24 mmol, 1.10 equiv.), K_2CO_3 (1.40 g, 10.1 mmol, 9.00 equiv.) and $Pd(PPh_3)_4$ (130 mg, 112 μmol, 0.10 equiv.) in 110 ml of THF/water (10:1). The product **S3** was isolated as a colorless solid (441 mg, 736 μmol, 66%).

$\boldsymbol{R_f}$ = 0.45 (SiO_2; PE/DCM 4:1).

^{1}H NMR (400 MHz, $CDCl_3$, 295 K): δ = 7.68 (s, 2H), 7.57 (s, 4H), 7.49 (s, 2H), 5.37 (s, 2H), 1.53 (s, 36H) ppm.

^{13}C{^{1}H} NMR (101 MHz, $CDCl_3$, 295 K): δ = 154.4, 143.8, 138.2, 136.6, 132.5, 125.8, 123.6, 120.3, 118.8, 34.6, 30.4 ppm.

IR (ATR): $\tilde{v}$ = 3632, 2954, 1433, 1417, 1387, 1360, 1316, 1236, 1220, 1180, 1145, 1109, 879, 836, 822, 808, 756, 708, 643, 630 cm^{-1}.

HRMS (MALDI$^+$, DCTB): m/z: calcd. for $C_{38}H_{46}O_2S_2$: 598.2934 $[M]^{\cdot+}$, found: 598.2916.

Mp: > 300 °C.

4,4'-(Dithieno[3,2-*f*:2',3'-*h*]quinoxaline-6,9-diyl)bis(2,6-di-*tert*-butylphenol) (S5)

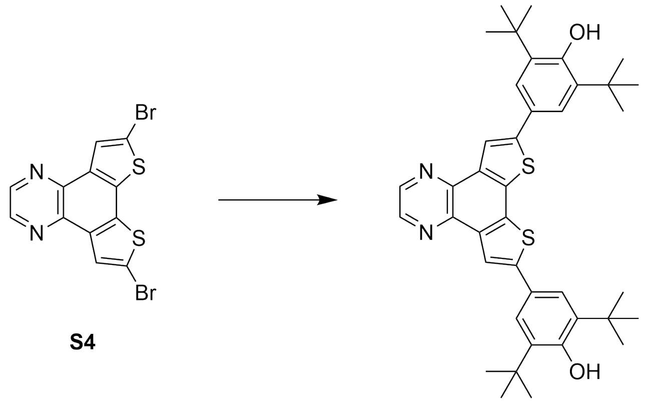

S4

S5

GP1 was applied to **S4** (200 mg, 500 μmol, 1.00 equiv.), **S1** (456 mg, 500 μmol, 1.00 equiv.), K_2CO_3 (622 mg, 4.50 mmol, 9.00 equiv.) and $Pd(PPh_3)_4$ (57.8 mg, 50.0 μmol, 0.10 equiv.) in 44 ml of THF/water (10:1). The product **S5** was isolated as a brown solid (170 mg, 261 μmol, 52%).

$\boldsymbol{R_f}$ = 0.28 (SiO_2; PE/EE 3:1).

^{1}H NMR (600 MHz, $CDCl_3$, 295 K): δ = 8.94 (s, 2H), 8.39 (s, 2H), 7.66 (s, 4H), 5.43 (s, 2H), 1.54 (s, 36H) ppm.

^{13}C{^{1}H} NMR (151 MHz, $CDCl_3$, 295 K): δ = 154.7, 145.4, 142.6, 138.7, 136.8, 135.3, 134.5, 125.4, 123.7, 117.8, 34.7, 30.4 ppm.

IR (ATR): $\tilde{\nu}$ = 3626, 2953, 2872, 1435, 1407, 1361, 1318, 1238, 1221, 1183, 1154, 1119, 1034, 872, 833, 703, 470 cm^{-1}.

HRMS (MALDI$^+$, DCTB): m/z: calcd. for $C_{40}H_{46}N_2O_2S_2$: 650.2995 $[M]^{\cdot+}$, found: 650.2981.

Mp: > 300 °C.

4,4'-(8,11-Bis((triisopropylsilyl)ethynyl)dithieno[3,2-*a*:2',3'-*c*]phenazine-2,5-diyl)bis(2,6-di-*tert*-butylphenol) (S7)

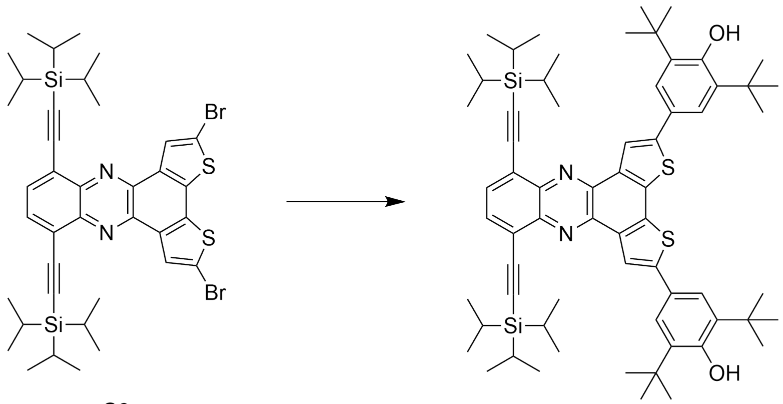

S6 **S7**

GP1 was applied to **S6** (100 mg, 123 μmol, 1.00 equiv.), **S1** (113 mg, 123 μmol, 1.00 equiv.), K_2CO_3 (153 mg, 1.11 mmol, 9.00 equiv.) and $Pd(PPh_3)_4$ (14.3 mg, 12.3 μmol, 0.10 equiv.) in 22 ml of THF/water (10:1). The product **S7** was isolated as a dark red solid (116 mg, 109 μmol, 89%).

$\boldsymbol{R_f}$ = 0.14 (SiO_2; PE/DCM 10:1).

^{1}H NMR (600 MHz, $CDCl_3$, 295 K): δ = 8.46 (s, 2H), 8.00 (s, 2H), 7.61 (s, 4H), 5.41 (s, 2H), 1.55 (s, 36H), 1.34-1.24 (m, 42H) ppm.

^{13}C{^{1}H} NMR (151 MHz, $CDCl_3$, 295 K): δ = 154.6, 145.0, 141.5, 140.2, 136.8, 135.7, 134.0, 125.8, 124.1, 119.3, 104.5, 99.7, 34.6, 30.4, 19.1, 11.6 ppm.

IR (ATR): $\tilde{\upsilon}$ = 3623, 2941, 2862, 2145, 1461, 1420, 1315, 1238, 1150, 1046, 996, 881, 839, 802, 715, 673, 591, 464 cm^{-1}.

HRMS ($MALDI^+$, DCTB): m/z: calcd. for $C_{66}H_{88}N_2O_2S_2Si_2$: 1060.5820 $[M]^+$, found: 1060.5810.

Mp: > 300 °C.

Crystal data: Plate-shaped, red crystals were obtained by diffusion of methanol into a chloroform solution of **S7**.

4,4'-(8,13-Bis((triisopropylsilyl)ethynyl)benzo[i]dithieno[3,2-*a*:2',3'-*c*]phenazine-2,5-diyl)bis(2,6-di-*tert*-butylphenol) (S9)

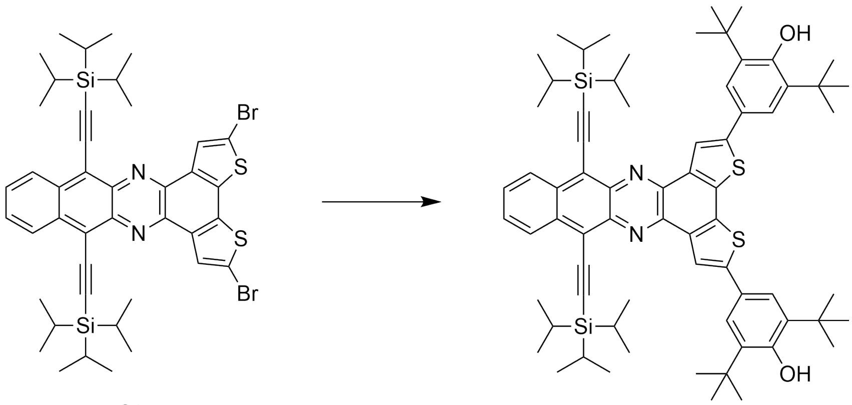

S8 **S9**

GP1 was applied to **S8** (150 mg, 174 μmol, 1.00 equiv.), **S1** (159 mg, 174 μmol, 1.00 equiv.), K_2CO_3 (217 mg, 1.57 mmol, 9.00 equiv.) and $Pd(PPh_3)_4$ (20.1 mg, 17.4 μmol, 0.10 equiv.) in 22 ml THF/water (10:1). The product **S9** was isolated as a dark green solid (172 mg, 155 μmol, 89%).

$\boldsymbol{R_f}$ = 0.11 (SiO_2; PE/DCM 10:1).

^{1}H NMR (600 MHz, $CDCl_3$, 295 K): δ = 8.86 – 8.83 (m, 2H), 8.49 (s, 2H), 7.72-7.69 (m, 2H), 7.61 (s, 4H), 5.42 (s, 2H), 1.56 (s, 36H), 1.35 (s, 42H) ppm.

^{13}C{^{1}H} NMR (151 MHz, $CDCl_3$, 295 K): δ = 154.6, 145.0, 141.5, 139.6, 136.8, 136.0, 135.7, 135.0, 127.8, 125.8, 124.1, 120.5, 119.7, 107.1, 103.4, 34.6, 30.5, 19.3, 11.7 ppm.

IR (ATR): $\tilde{v}$ = 3618, 2942, 2863, 1418, 1317, 1237, 1046, 880, 845, 771, 763, 719, 673, 561, 481, 471, 458, 443 cm^{-1}.

HRMS (MALDI$^-$, DCTB): *m/z*: calcd. for $C_{70}H_{90}N_2O_2S_2Si_2$: 1109.5909 $[M-H]^{\cdot-}$, found: 1109.5914.

Mp: > 300 °C.

4,4'-(Benzo[2,1-*b*:3,4-*b*']dithiophene-2,7-diylidene)bis(2,6-di-*tert*-butylcyclohexa-2,5-dien-1-one) (BT-1)

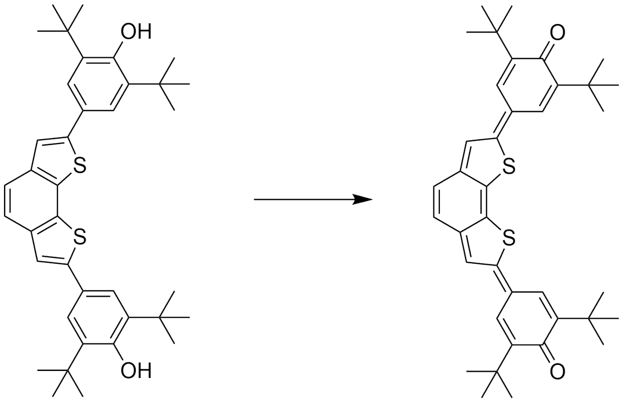

S3 **BT-1**

GP2 was applied to **S3** (30.2 mg, 50.4 µmol, 1.00 equiv.), $K_3[Fe(CN)_6]$ (33.2 mg, 101 µmol, 2.00 equiv.) in 60 ml of a 1:1 mixture of THF and aqueous KOH (0.1 M) under argon atmosphere. The product **BT-1** was obtained as a dark purple solid (30.0 mg, 50.3 µmol, 100%).

$\boldsymbol{R_f}$ = 0.15 (SiO_2; PE/DCM 3:1).

^{1}H NMR (600 MHz, CD_2Cl_2:CS_2 1:1, 295 K): δ = 7.51 (s, 2H), 1.40 (s, 36H) ppm.

^{1}H NMR (600 MHz, CD_2Cl_2:CS_2 1:1, 238 K): δ = 7.77 (s, 2H), 7.54 (s, 4H), 7.30 (s, 2H), 1.35 (s, 36H) ppm.

^{13}C{^{1}H} NMR (151 MHz, CD_2Cl_2:CS_2 1:1, 295 K): δ = 124.9 ppm.

IR (ATR): $\tilde{\nu}$ = 2953, 1563, 1482, 1449, 1383, 1354, 1319, 1254, 1094, 1023, 984, 930, 890, 846, 813, 703, 655, 601, 529, 495 cm^{-1}.

HRMS (MALDI+, DCTB): m/z: calcd. for $C_{38}H_{44}O_2S_2$: 596.2777 $[M]^{\cdot+}$, found: 596.2777.

Mp: 225 °C.

4,4'-(Dithieno[3,2-*f*:2',3'-*h*]quinoxaline-6,9-diylidene)bis(2,6-di-*tert*-butylcyclohexa-2,5-dien-1-one) (BT-2)

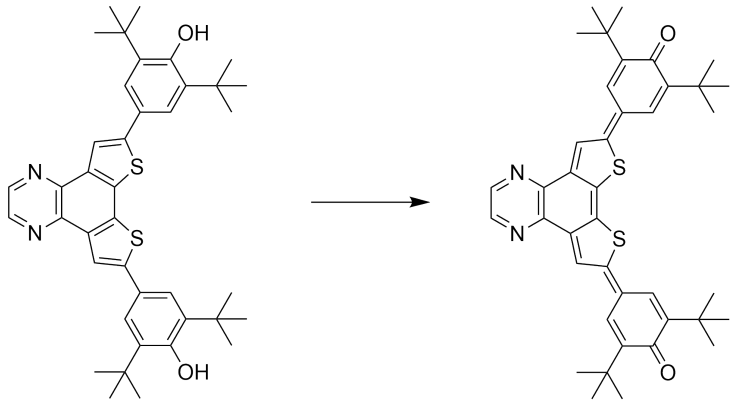

S5 **BT-2**

GP2 was applied to **S5** (40.5 mg, 62.2 µmol, 1.00 equiv.), $K_3[Fe(CN)_6]$ (41.0 mg, 124 µmol, 2.00 equiv.) in 70 ml of a 1:1 mixture of THF and aqueous KOH (0.1 M) under argon atmosphere. The product **BT-2** was obtained as a dark green solid (40.0 mg, 61.6 µmol, 99%).

$\boldsymbol{R_f}$ = 0.56 (SiO_2; PE/EE 1:1).

^{1}H NMR (600 MHz, CD_2Cl_2:CS_2 1:1, 295 K): δ = 8.87 (s, 2H), 1.42 (s, 36H) ppm. **^{1}H NMR** (600 MHz, CD_2Cl_2:CS_2 1:1, 238 K): δ = 8.83 (s, 2H), 8.45 (s, 2H), 7.57 (s, 2H), 7.24 (s, 2H), 1.38 (s, 18H), 1.36 (s, 18H) ppm.
^{13}C^{1}H NMR (151 MHz, CD_2Cl_2:CS_2 1:1, 295 K): δ = 145.2, 141.0 ppm.

IR (ATR): $\tilde{\nu}$ = 2951, 1568, 1483, 1451, 1387, 1358, 1254, 1199, 1130, 1085, 1025, 990, 925, 889, 840, 812, 710, 643, 528, 452 cm^{-1}.
HRMS (MALDI+, DCTB): m/z: calcd. for $C_{40}H_{44}N_2O_2S_2$: 648.2839 $[M]^{\cdot+}$, found: 648.2839.
Mp: 236 °C.

4,4'-(8,11-Bis((triisopropylsilyl)ethynyl)dithieno[3,2-*a*:2',3'-*c*]phenazine-2,5-diylidene)bis(2,6-di-*tert*-butylcyclohexa-2,5-dien-1-one) (BT-3)

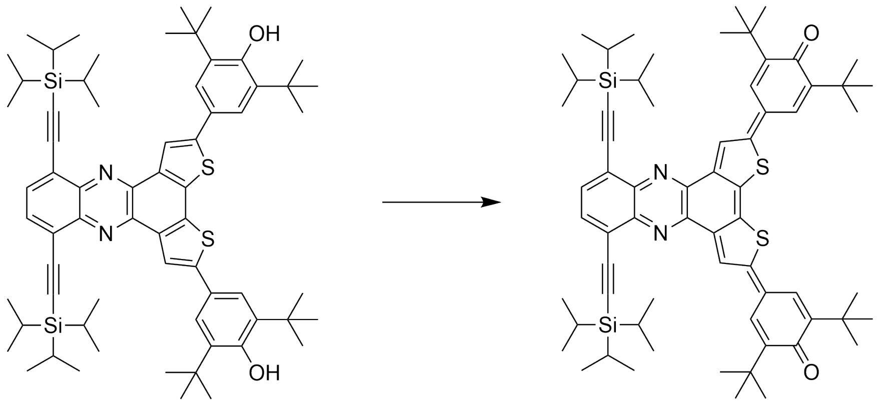

S7 **BT-3**

GP2 was applied to **S7** (20.0 mg, 18.8 μmol, 1.00 equiv.), $K_3[Fe(CN)_6]$ (12.4 mg, 37.7 μmol, 2.00 equiv.) in 20 ml of a 1:1 mixture of THF and aqueous KOH (0.1 M) under argon atmosphere. The product **BT-3** was obtained as dark green solid (19.5 mg, 18.8 μmol, 99%).

$\boldsymbol{R_f}$ = 0.25 (SiO_2; PE/DCM 7:3).

^{1}H NMR (600 MHz, $CDCl_3$, 295 K): δ = 8.02 (s, 2H), 1.42 (s, 36H), 1.34-1.18 (m, 42H) ppm.

^{1}H NMR (600 MHz, CD_2Cl_2, 295 K): δ = 8.01 (s, 2H), 1.41 (s, 36H), 1.29-1.25 (m, 42H) ppm.

^{1}H NMR (600 MHz, CD_2Cl_2, 238 K): δ = 8.38 (s, 2H), 7.81 (s, 2H), 7.47 (s, 2H), 7.18 (s, 2H), 1.40 (s, 18H), 1.33 (s, 18H), 1.28-1.20 (m, 42H) ppm.

^{13}C{^{1}H} NMR (151 MHz, $CDCl_3$, 295 K): δ = 142.2, 141.2, 135.7, 124.3, 103.7, 101.5, 19.1, 11.5 ppm.

IR (ATR): $\tilde{v}$ = 2942, 2888, 2862, 1567, 1451, 1385, 1358, 1255, 1155, 1137, 1085, 1036, 1023, 981, 881, 847, 801, 784, 717, 671, 661, 636, 626, 587, 530, 501, 450, 436 cm^{-1}.

HRMS ($MALDI^-$, DCTB): m/z: calcd. for $C_{66}H_{86}N_2O_2S_2Si_2$: 1058.5664 $[M]^{\cdot+}$, found: 1058.5665.

Mp: > 300 °C.

Crystal data: Prism-shaped, blue/bronze crystals were obtained by diffusion of methanol into a chloroform solution of **BT-3**.

4,4'-(8,13-Bis((triisopropylsilyl)ethynyl)benzo[i]dithieno[3,2-*a*:2',3'-*c*]phenazine-2,5-diylidene)bis(2,6-di-*tert*-butylcyclohexa-2,5-dien-1-one) (BT-4)

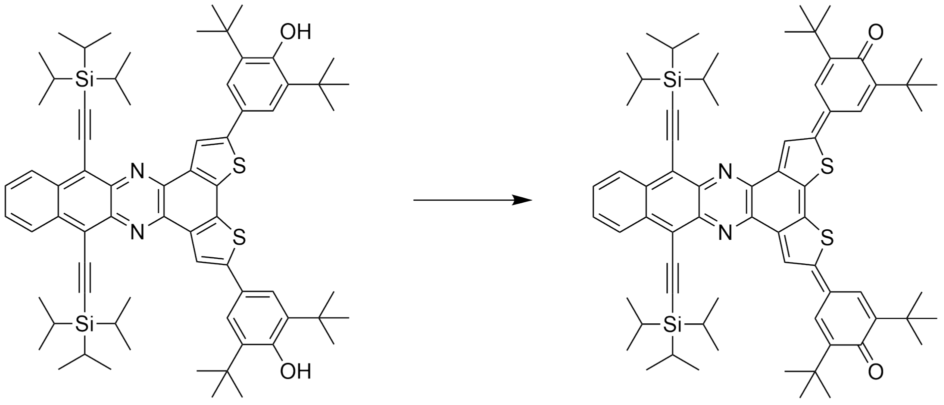

S9 **BT-4**

GP2 was applied to **S9** (50.0 mg, 45.0 μmol, 1.00 equiv.), $K_3[Fe(CN)_6]$ (29.6 mg, 90.0 μmol, 2.00 equiv.) in 50 ml of a 1:1 mixture of THF and aqueous KOH (0.1 M) under argon atmosphere. The product **BT-4** was obtained as dark green solid (49.0 mg, 44.2 μmol, 98%).

$\boldsymbol{R_f}$ = 0.44 (SiO_2; PE/DCM 1:1).

^{1}H NMR (700 MHz, $CDCl_3$, 295 K): δ = 8.83 – 8.82 (m, 2H), 7.77-7.76 (m, 2H), 1.43 (s, 36H), 1.39-1.27 (m, 42H) ppm.

^{1}H NMR (600 MHz, $CDCl_3$, 235 K): δ = 8.81 – 8.78 (m, 2H), 8.66 (s, 2H), 7.82-7.78 (m, 2H), 7.63 (s, 2H), 7.39 (s, 2H), 1.43-1.37 (m, 36H), 1.32-1.25 (m, 42H) ppm.

^{13}C{^{1}H} NMR (176 MHz, $CDCl_3$, 295 K): δ = 142.0, 139.6, 135.8, 128.9, 127.9, 121.2, 108.9, 102.6, 19.2, 11.7 ppm.

IR (ATR): $\tilde{v}$ = 2948, 2923, 2864, 2358, 2323, 1646, 1575, 1569, 1507, 1456, 1386, 1359, 1138, 1087, 1043, 982, 883, 846, 762, 720, 668, 484, 406 cm^{-1}.

HRMS ($MALDI^-$, DCTB): m/z: calcd. for $C_{70}H_{88}N_2O_2S_2Si_2$: 1108.5831 $[M]^{\cdot+}$, found: 1108.5823.

Mp: > 300 °C.

Crystal data: Needle-shaped, green crystals were obtained by diffusion of methanol into a chloroform solution of **BT-4**.

3. VT-NMR

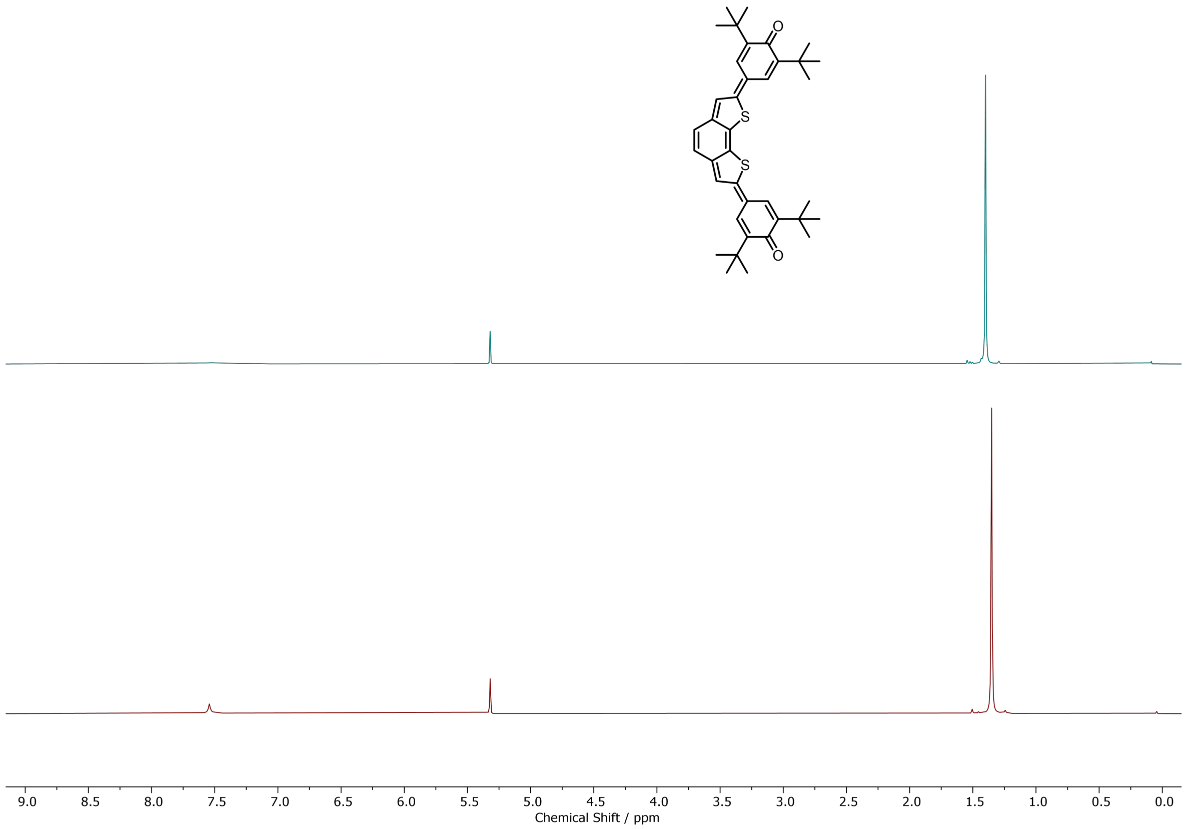

Figure S1. ^{1}H NMR spectra (600 MHz, CD_2Cl_2:CS_2 1:1) of **BT-1** at 295 K (top, green) and at 238 K (bottom, red).

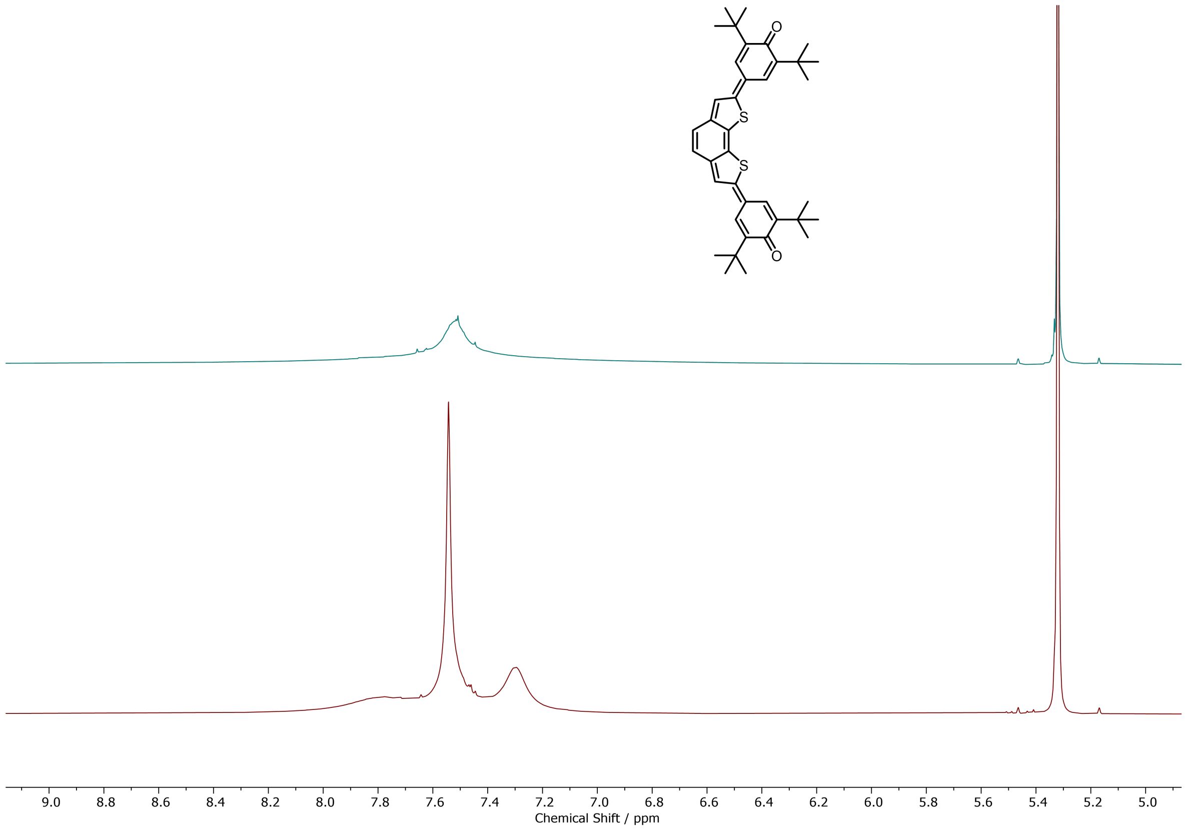

Figure S2. ^{1}H NMR spectra (600 MHz, CD_2Cl_2:CS_2 1:1) of **BT-1** in the region from 5.00 – 9.00 ppm at 295 K (top, green) and at 238 K (bottom, red).

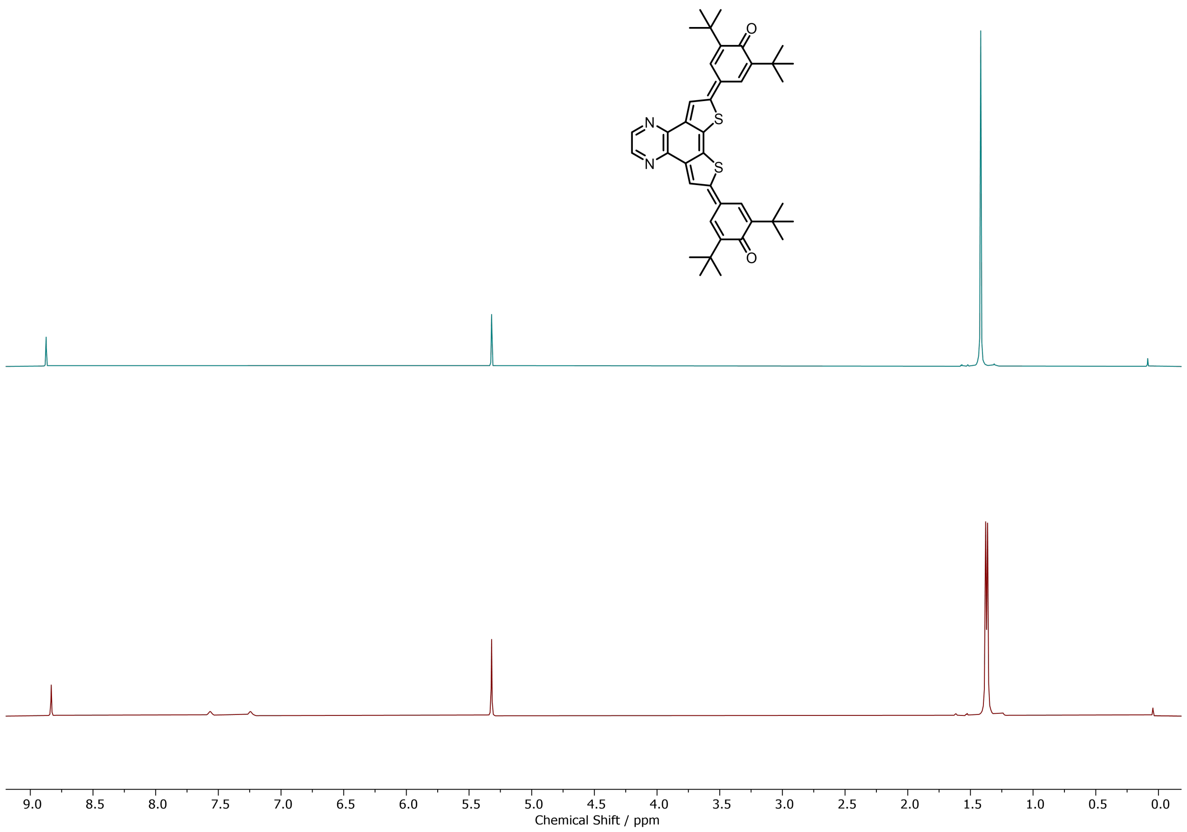

Figure S3. ^{1}H NMR spectra (600 MHz, CD_2Cl_2:CS_2 1:1) of **BT-2** at 295 K (top, green) and at 238 K (bottom, red).

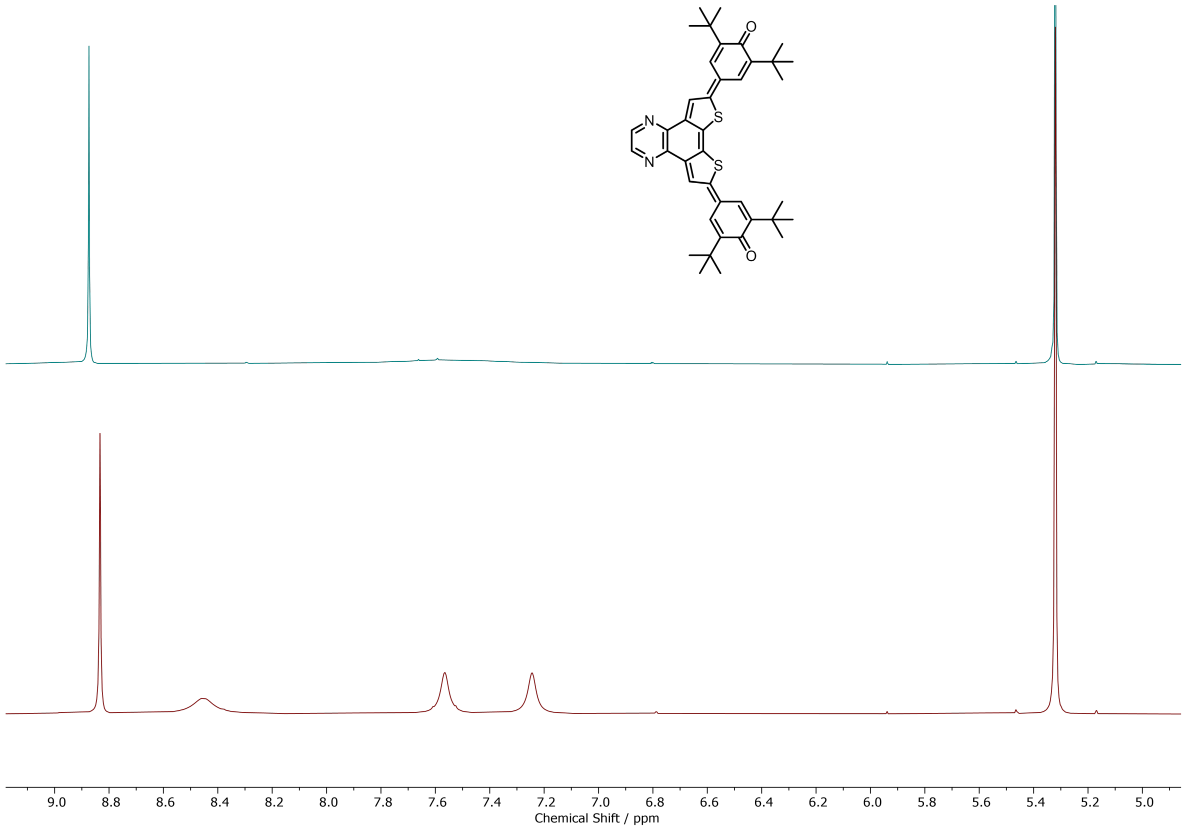

Figure S4. ^{1}H NMR spectra (600 MHz, CD_2Cl_2:CS_2 1:1) of **BT-2** in the region from 5.00 – 9.00 ppm at 295 K (top, green) and at 238 K (bottom, red).

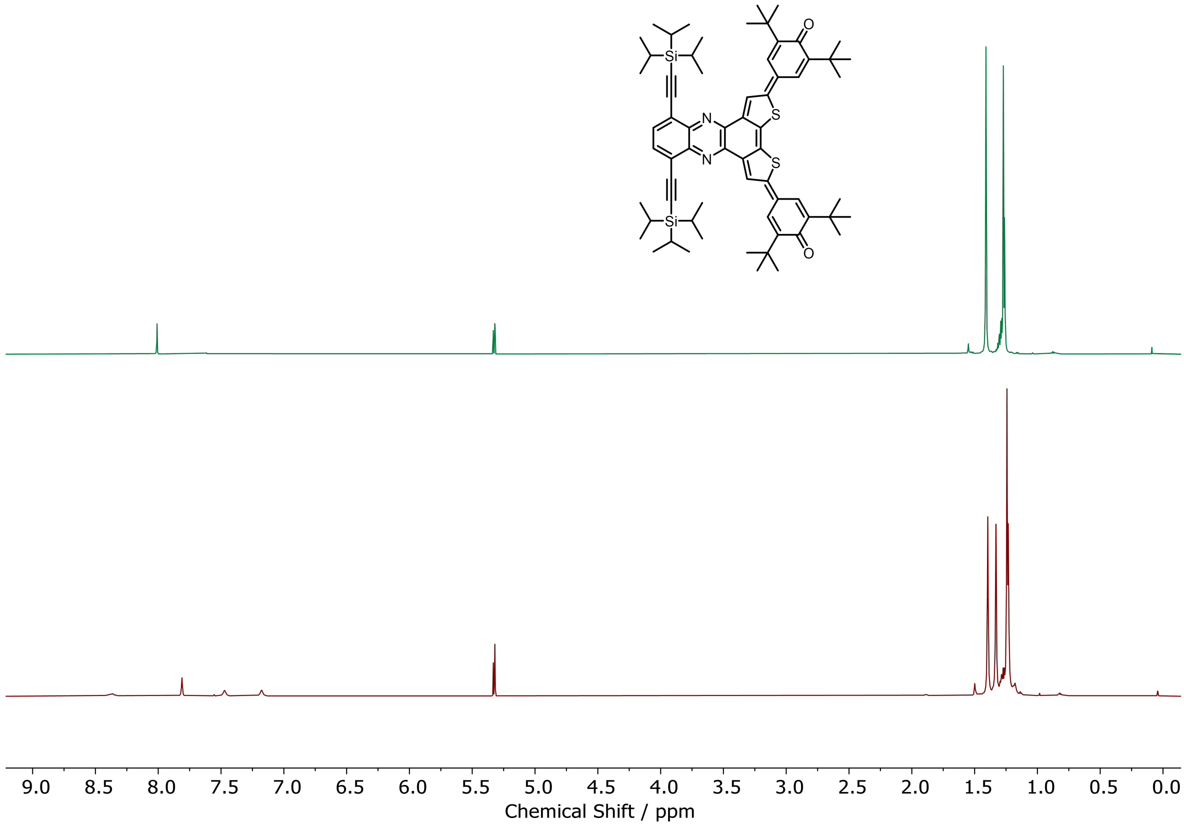

Figure S5. ^{1}H NMR spectra (600 MHz, CD_2Cl_2) of **BT-3** at 295 K (top, green) and at 238 K (bottom, red).

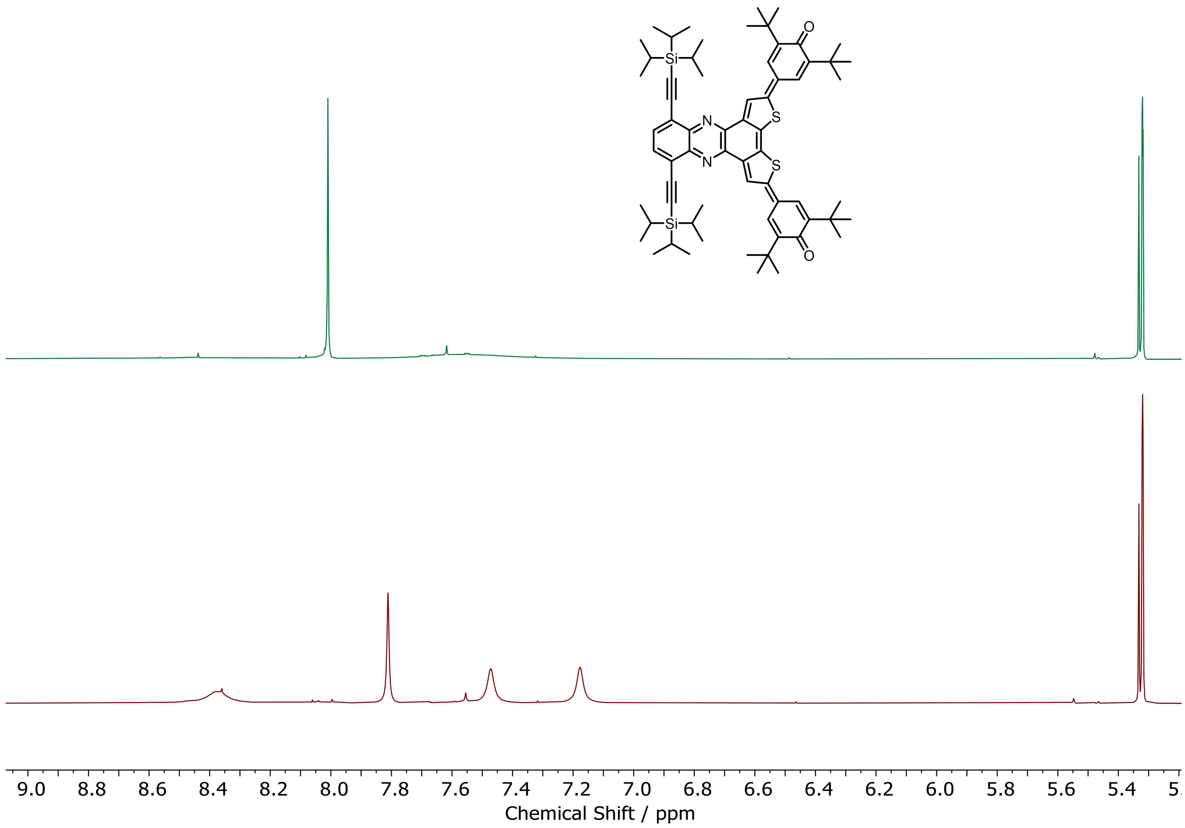

Figure S6. ^{1}H NMR spectra (600 MHz, CD_2Cl_2) of **BT-3** in the region from 5.30 – 9.00 ppm at 295 K (top, green) and at 238 K (bottom, red).

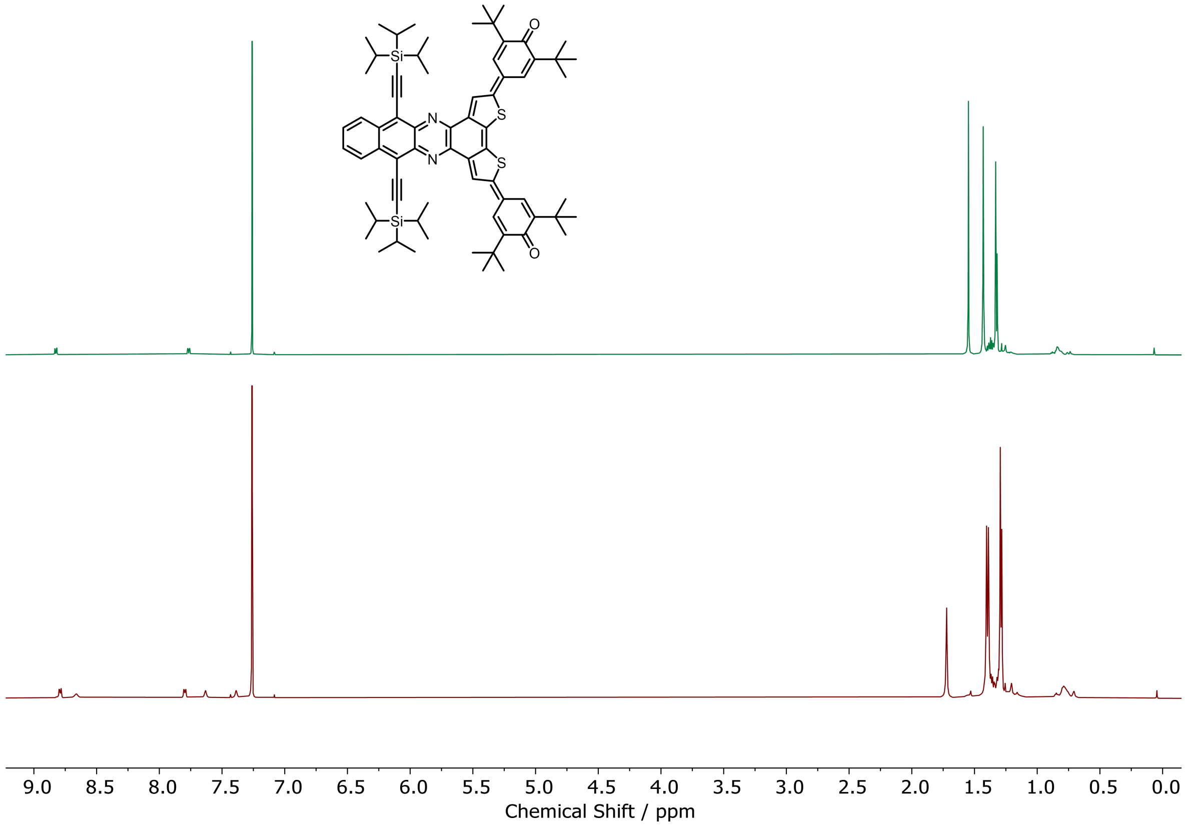

Figure S7. ^{1}H NMR spectra (600 MHz, $CDCl_3$) of **BT-4** at 295 K (top, green) and at 235 K (bottom, red).

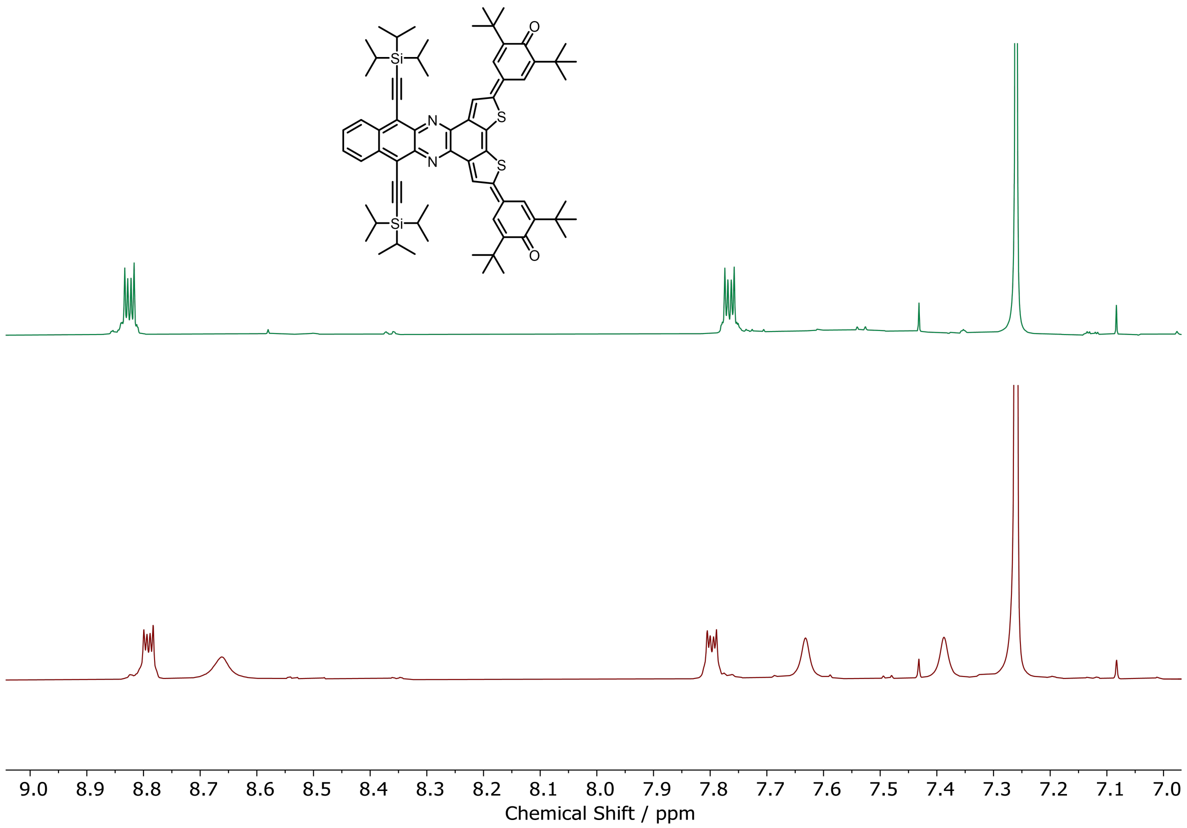

Figure S8. ^{1}H NMR spectra (600 MHz, $CDCl_3$) of **BT-4** in the region from 7.0 – 9.0 ppm at 295 K (top, green) and at 235 K (bottom, red).

4. NMR Spectroscopy

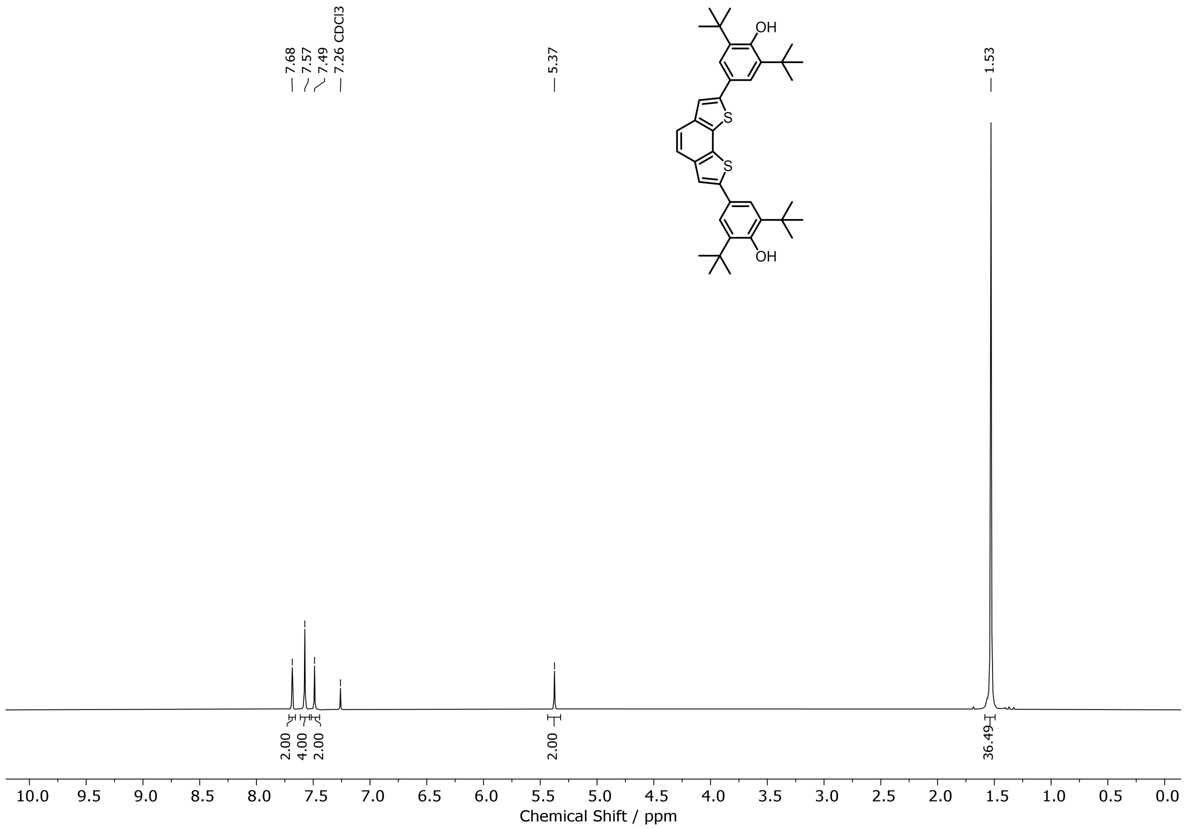

Figure S9. ^{1}H NMR spectrum (400 MHz, $CDCl_3$, 295 K) of **S3**.

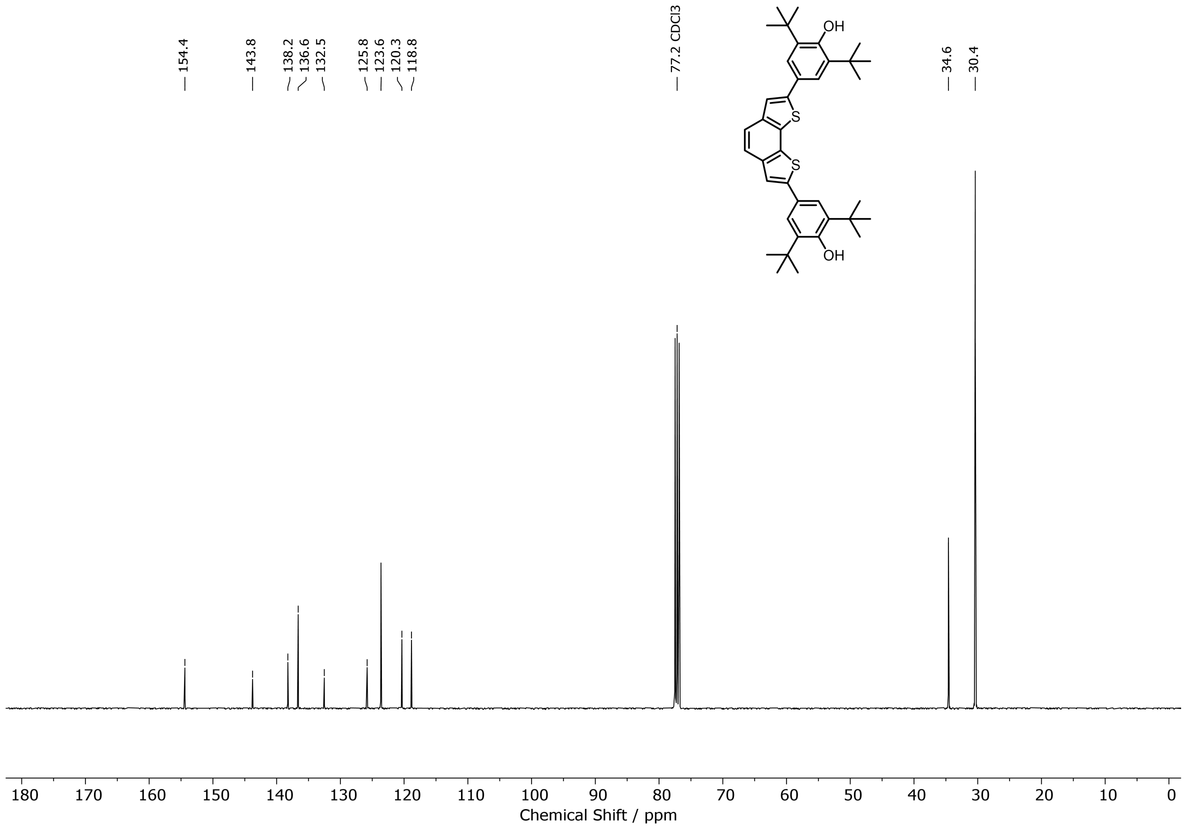

Figure S10. $^{13}C\{^{1}H\}$ NMR spectrum (101 MHz, $CDCl_3$, 295 K) of **S3**.

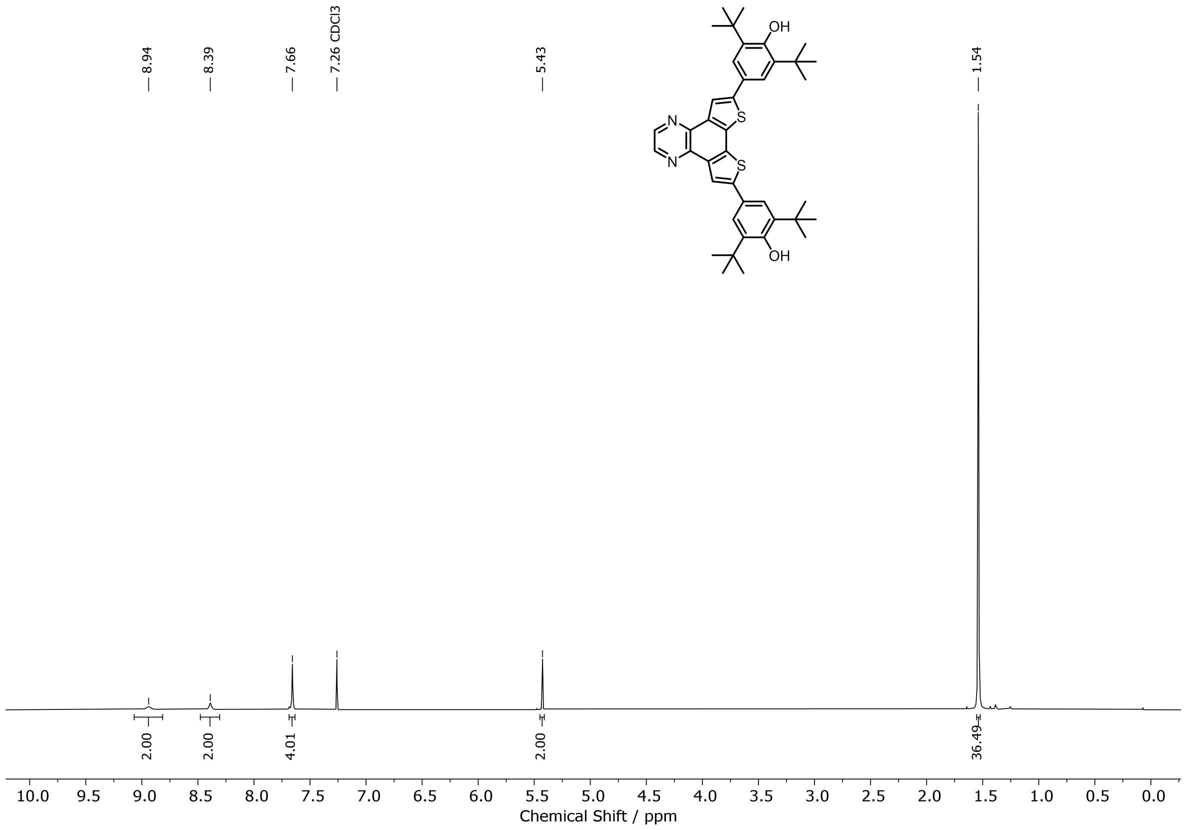

Figure S11. ^{1}H NMR spectrum (600 MHz, $CDCl_3$, 295 K) of **S5**.

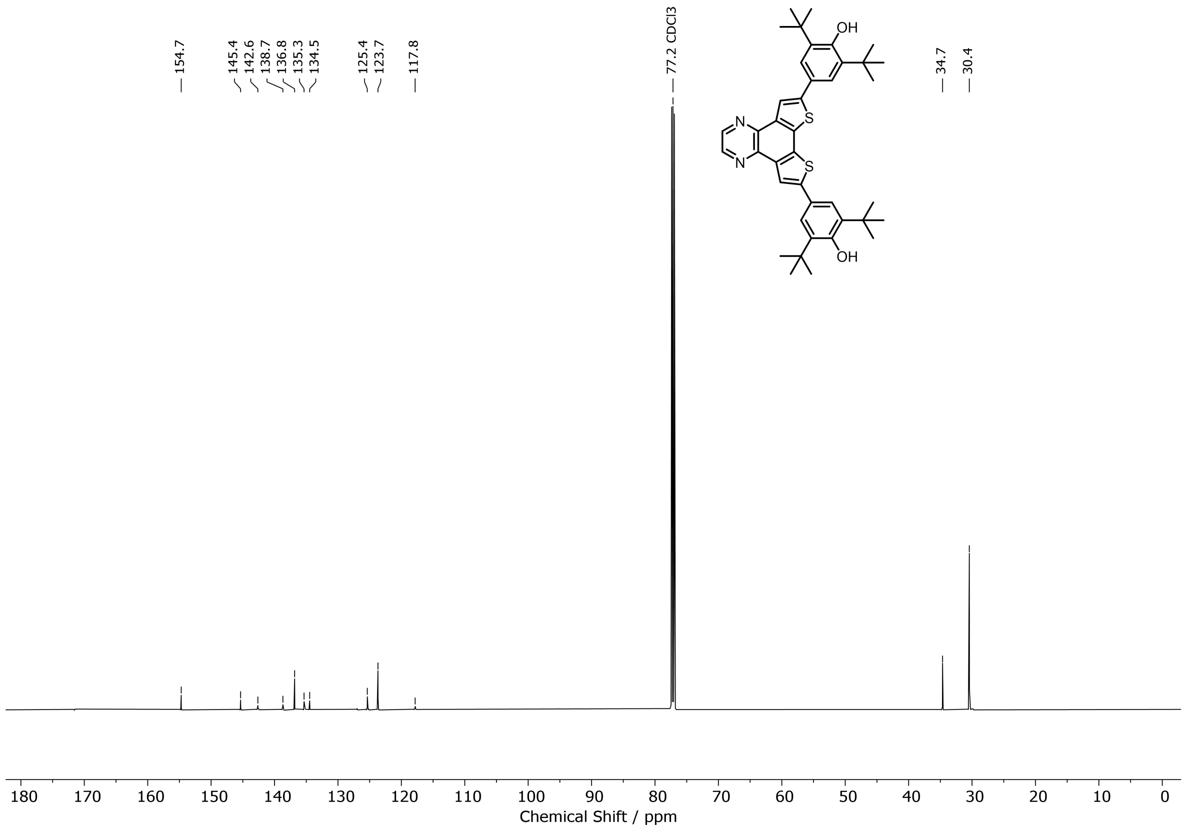

Figure S12. $^{13}C\{^{1}H\}$ NMR spectrum (151 MHz, $CDCl_3$, 295 K) of **S5**.

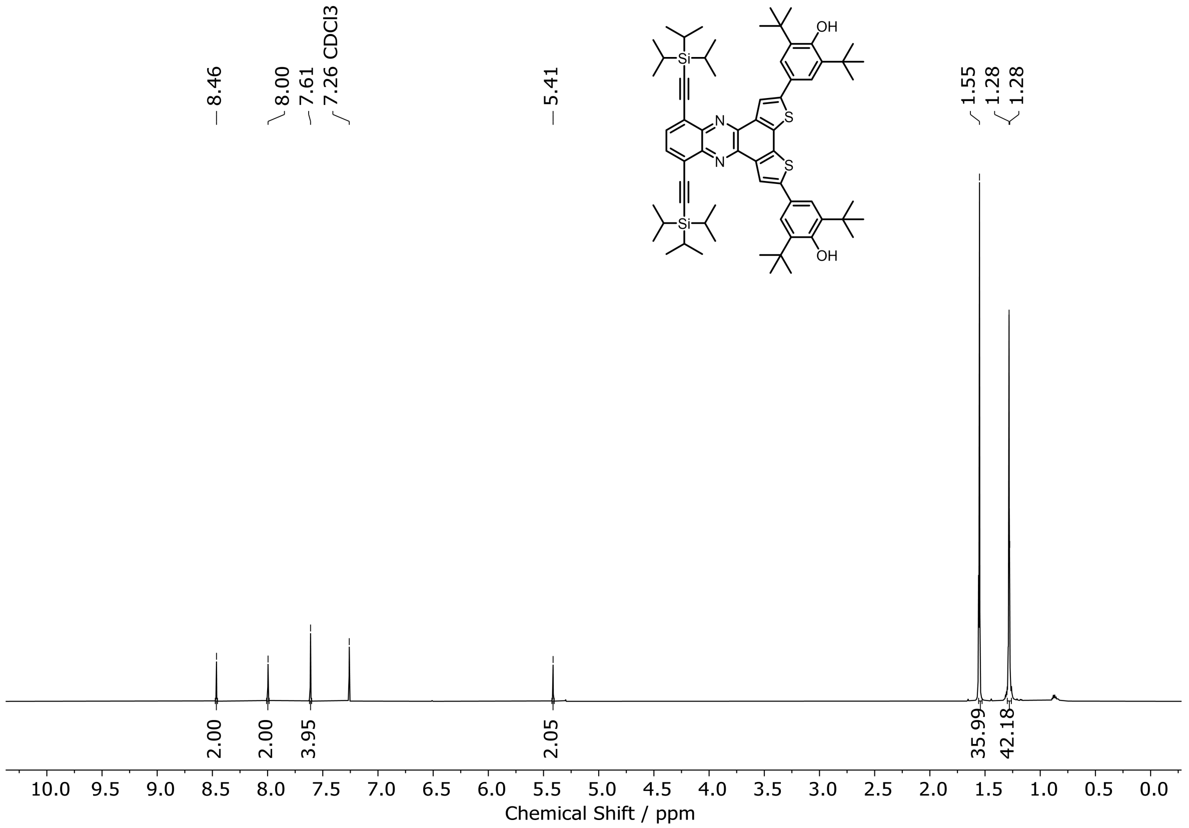

Figure S13. ^{1}H NMR spectrum (600 MHz, $CDCl_3$, 295 K) of **S7**.

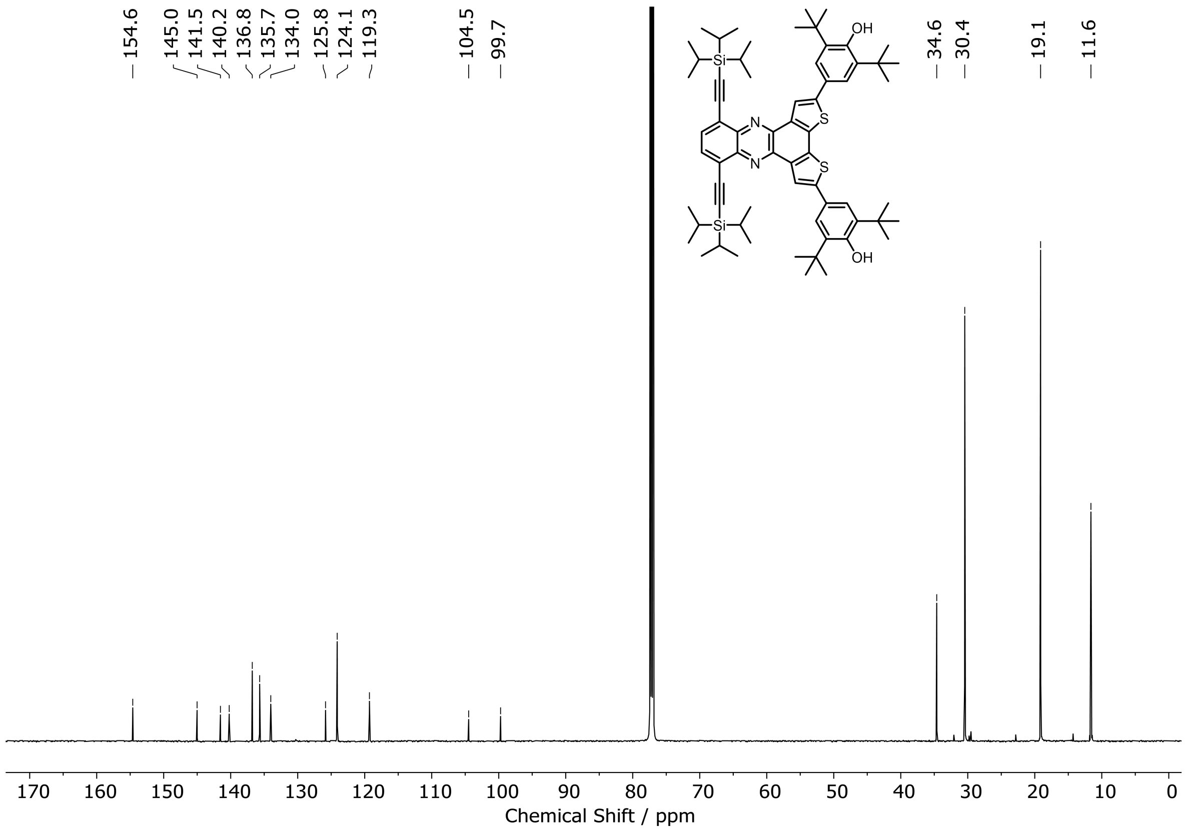

Figure S14. $^{13}C\{^{1}H\}$ NMR spectrum (151 MHz, $CDCl_3$, 295 K) of **S7**.

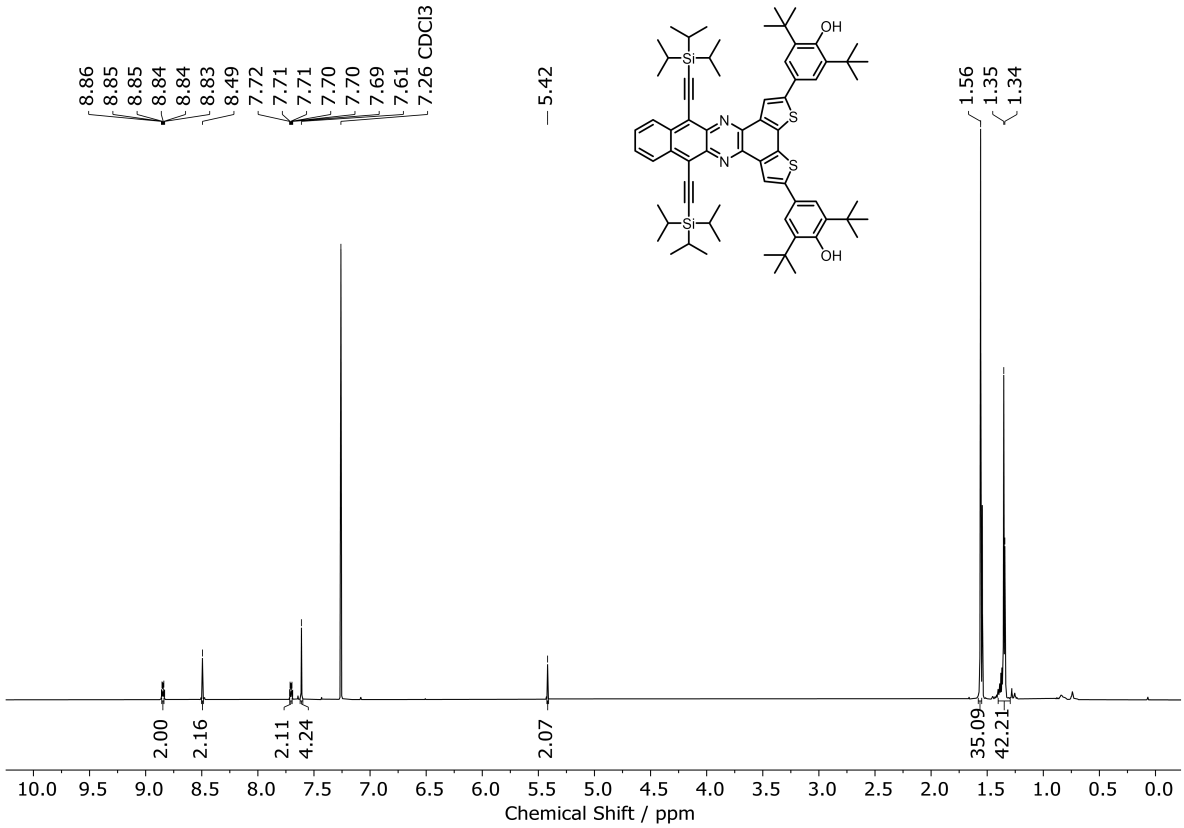

Figure S15. ^{1}H NMR spectrum (600 MHz, $CDCl_3$, 295 K) of **S9**.

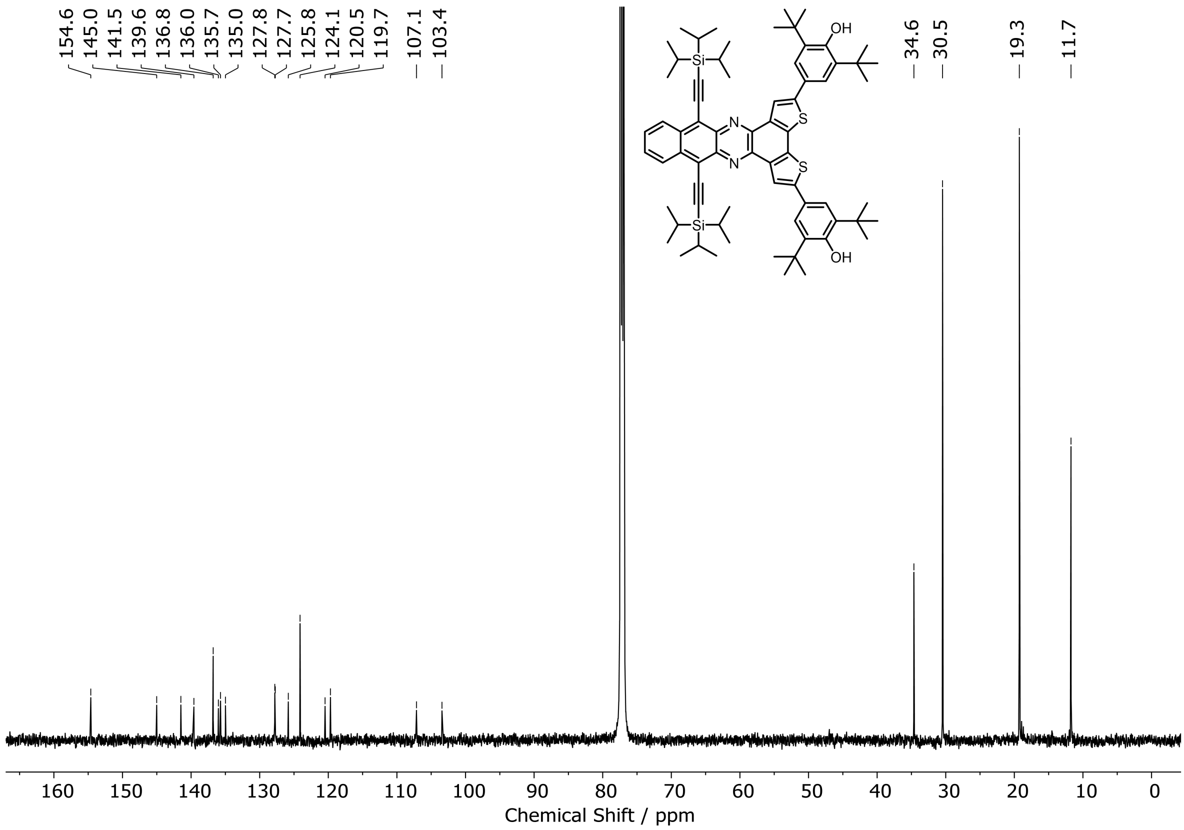

Figure S16. $^{13}C\{^{1}H\}$ NMR spectrum (151 MHz, $CDCl_3$, 295 K) of **S9**.

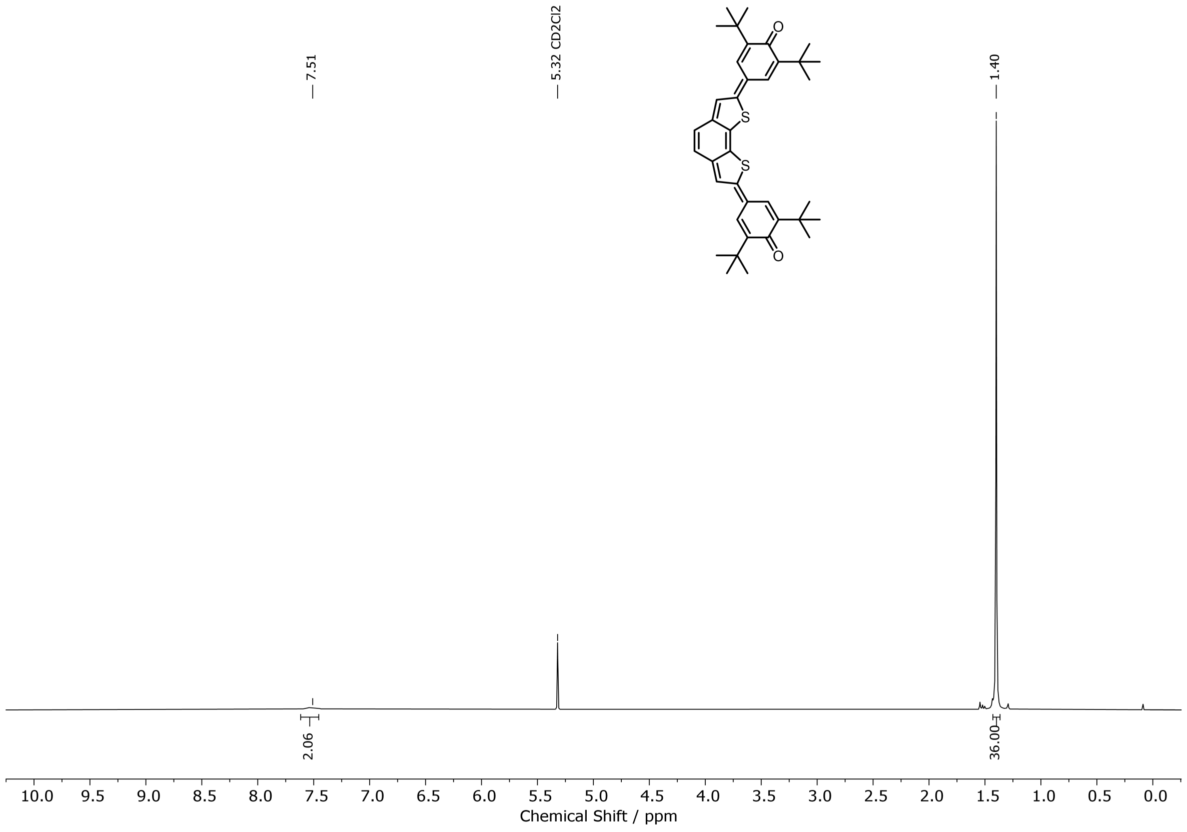

Figure S17. ^{1}H NMR spectrum (600 MHz, CD_2Cl_2:CS_2 1:1, 295 K) of **BT-1**.

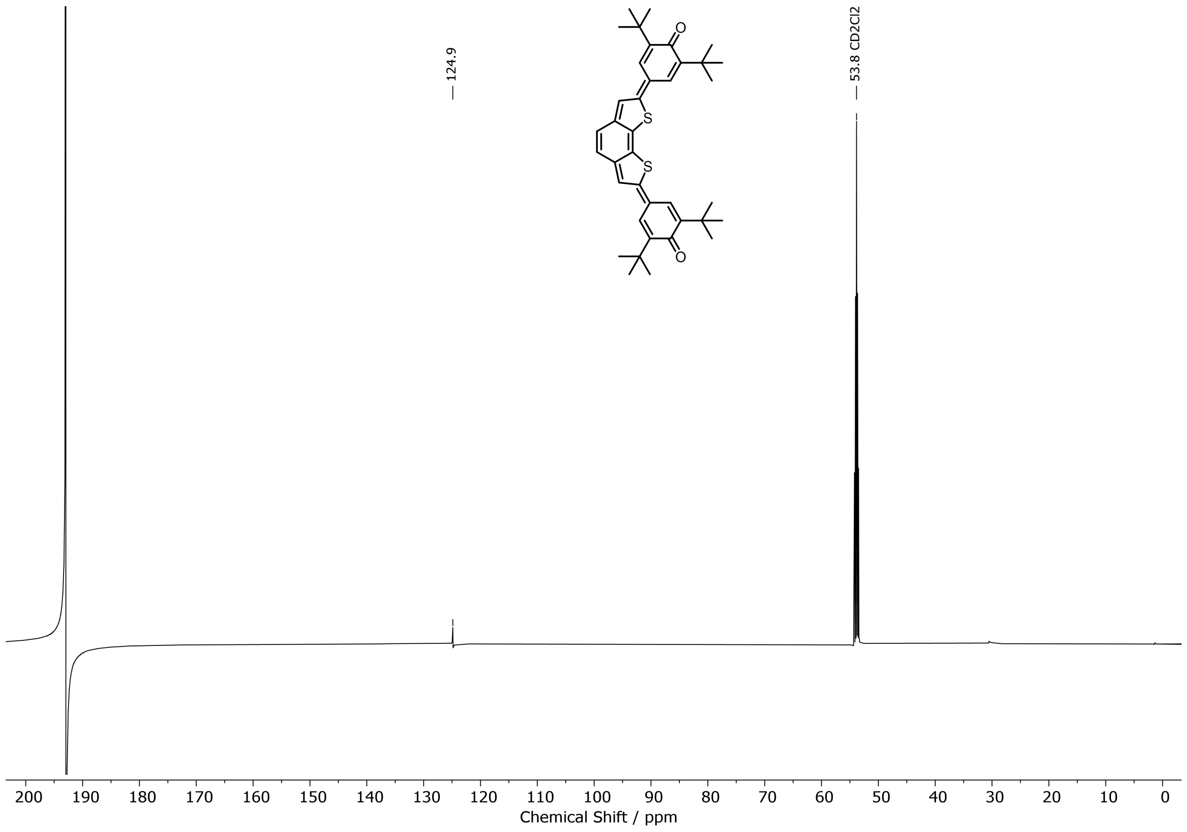

Figure S18. $^{13}C\{^{1}H\}$ NMR spectrum (151 MHz, CD_2Cl_2:CS_2 1:1, 295 K) of **BT-1**.

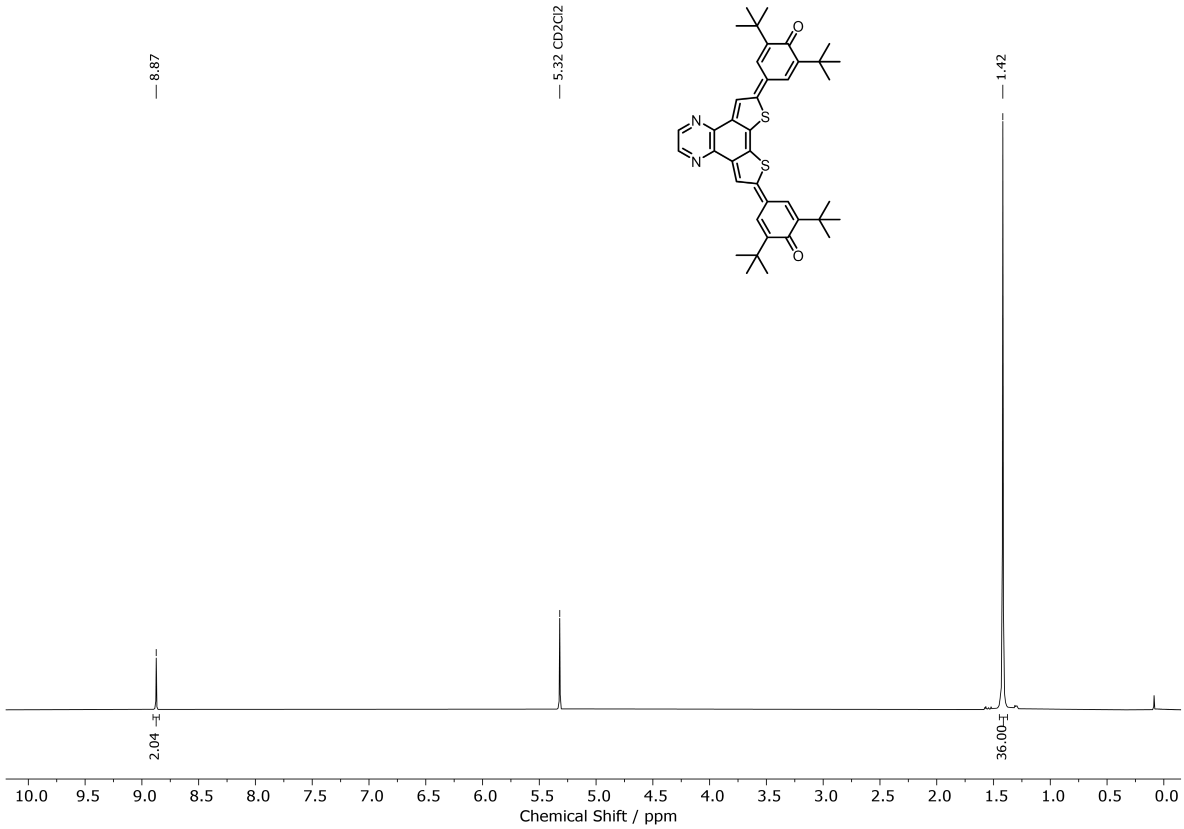

Figure S19. ^{1}H NMR spectrum (600 MHz, CD_2Cl_2:CS_2 1:1, 295 K) of **BT-2**.

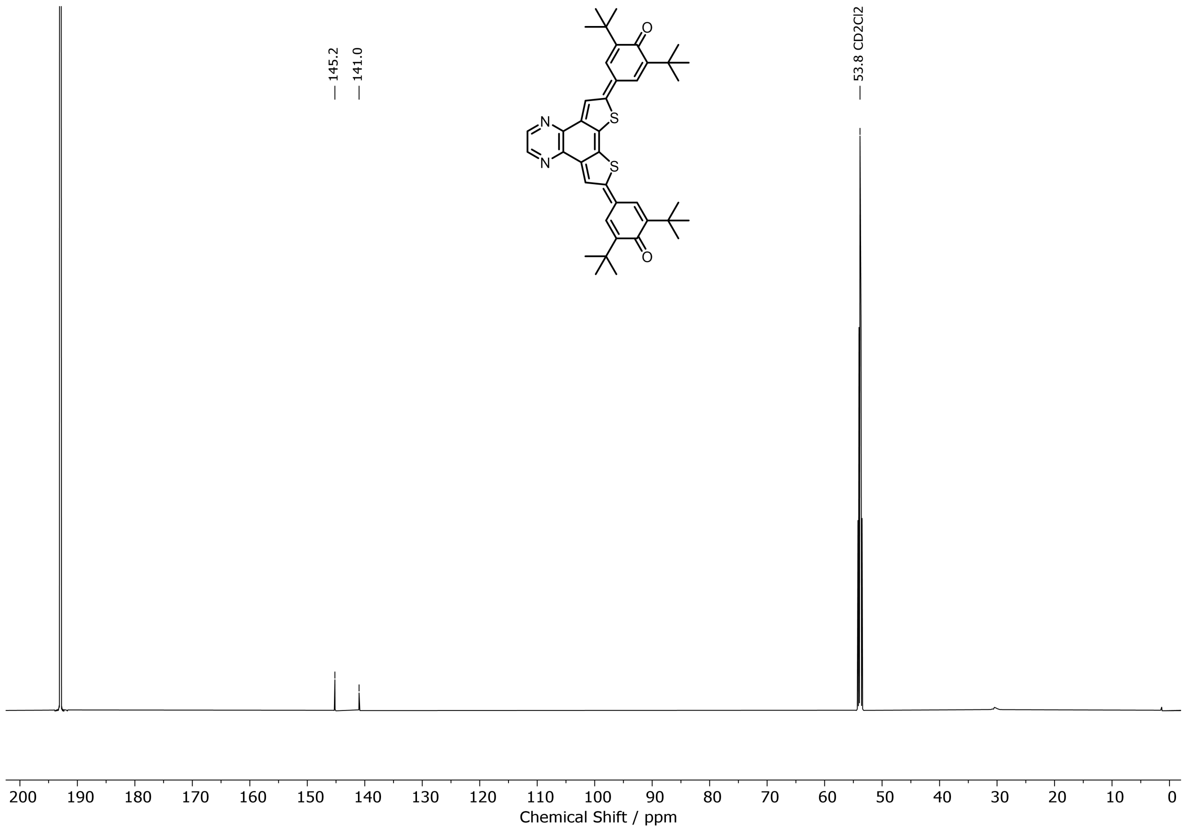

Figure S20. $^{13}C\{^{1}H\}$ NMR spectrum (151 MHz, CD_2Cl_2:CS_2 1:1, 295 K) of **BT-2**.

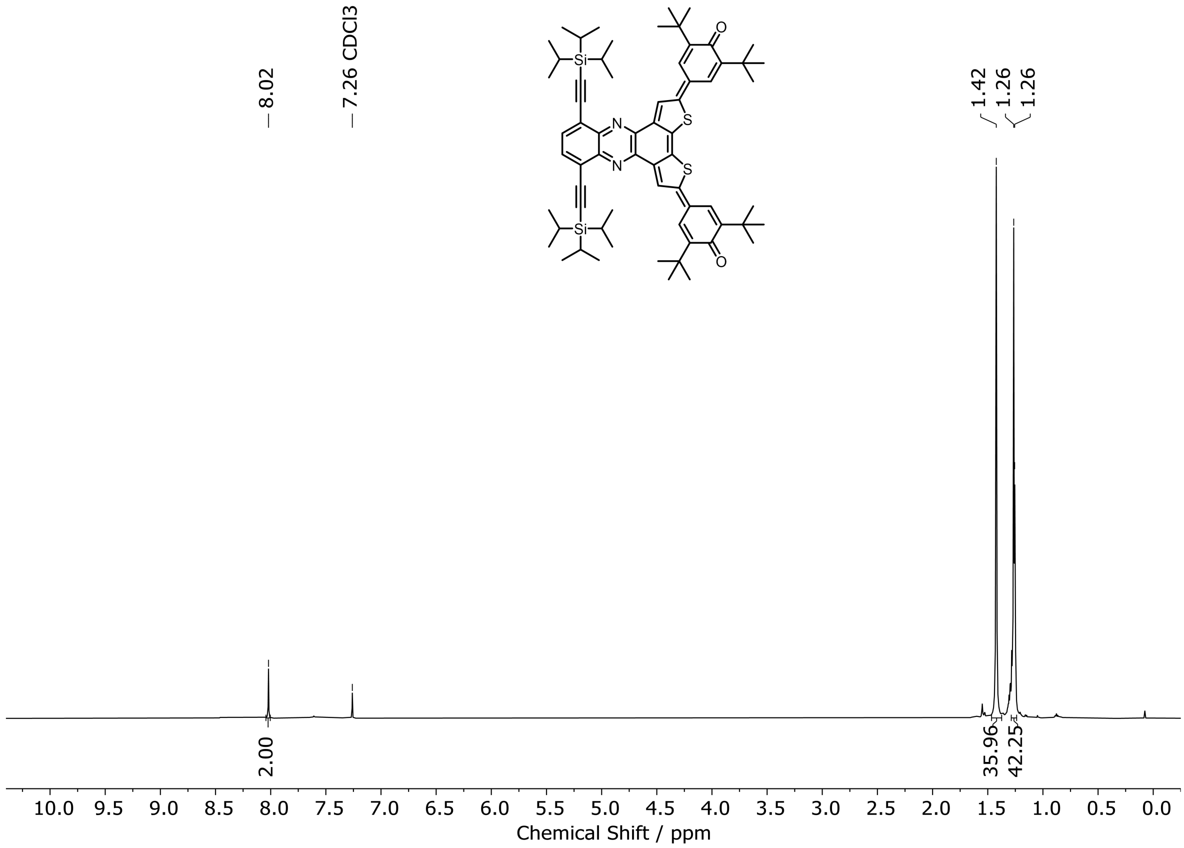

Figure S21. ^{1}H NMR spectrum (600 MHz, $CDCl_3$, 295 K) of **BT-3**.

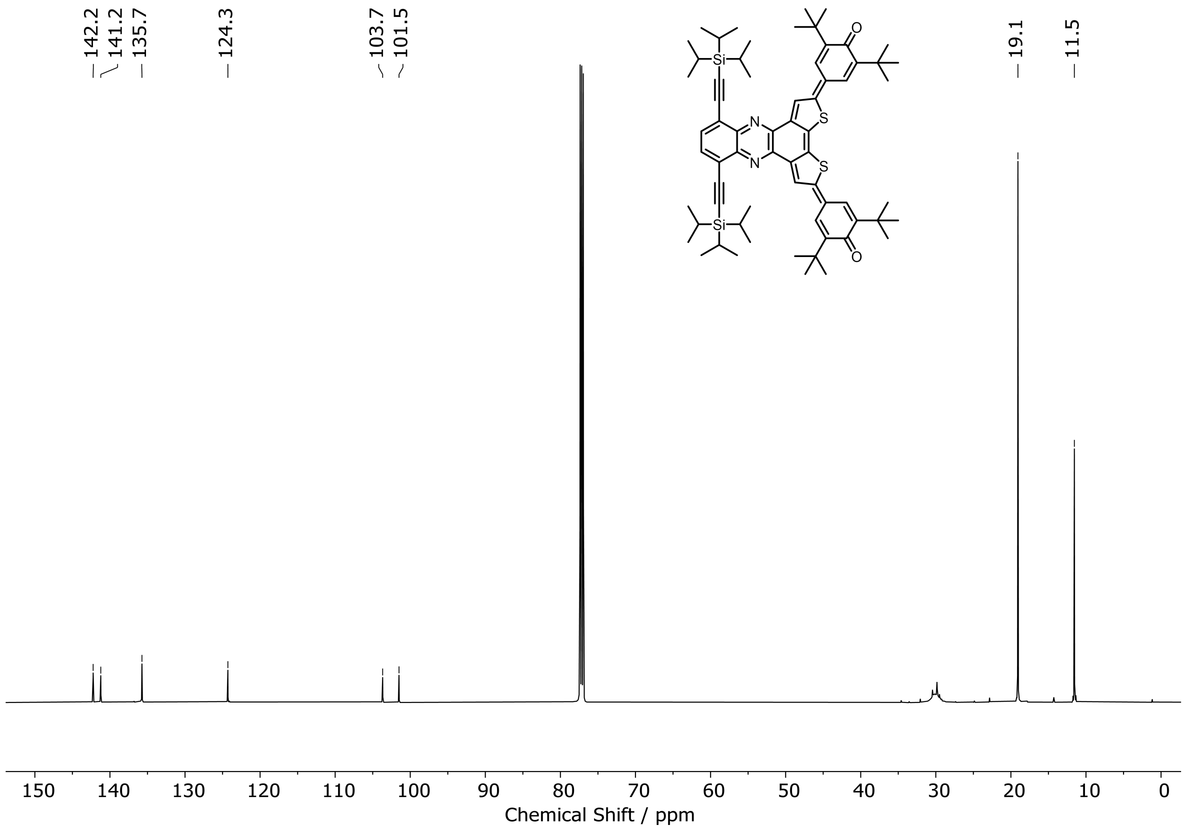

Figure S22. ^{13}C{^{1}H} NMR spectrum (151 MHz, $CDCl_3$, 295 K) of **BT-3**.

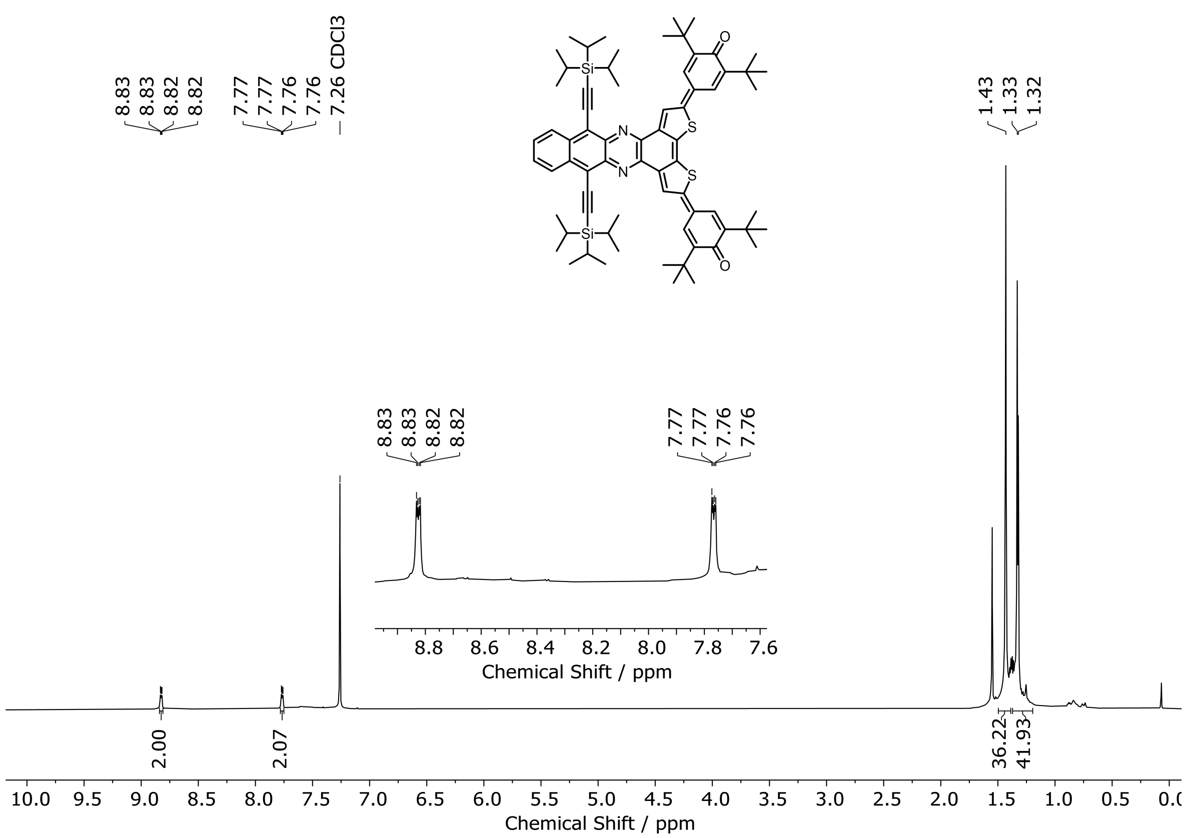

Figure S23. ^{1}H NMR spectrum (700 MHz, $CDCl_3$, 295 K) of **BT-4**.

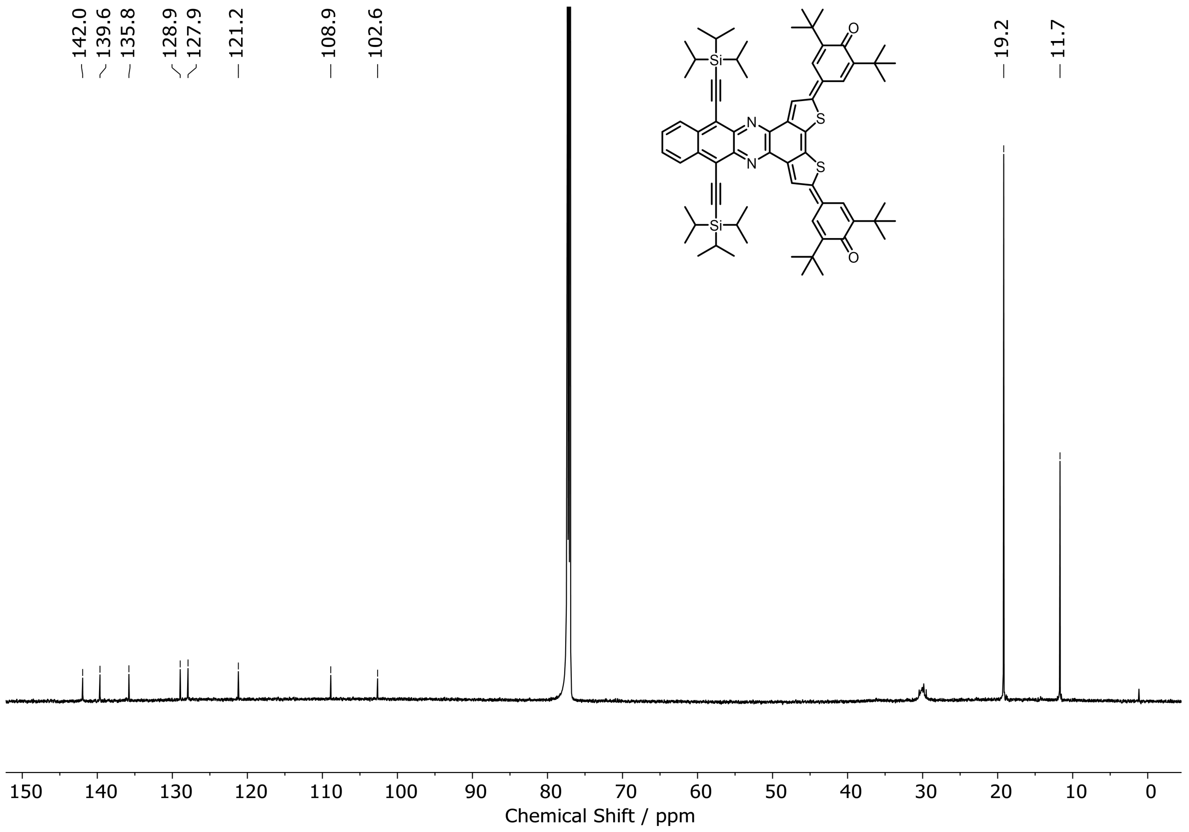

Figure S24. ^{13}C{^{1}H} NMR spectrum (176 MHz, $CDCl_3$, 295 K) of **BT-4**.

5. Crystallographic Data

Table S1. Crystal structure, crystal data and structure refinement for **S7** (CCDC 2411868).

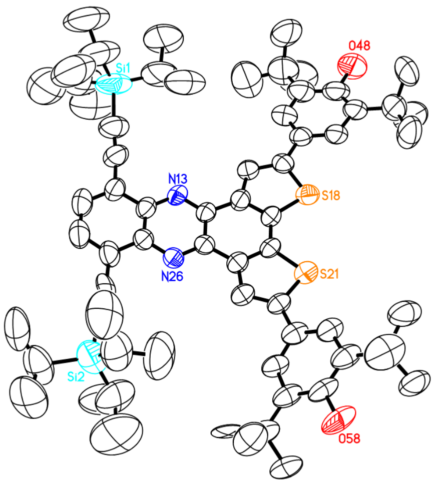

Empirical formula	$C_{66}H_{88}N_2O_2S_2Si_2$	
Formula weight	1061.68	
Temperature	200(2) K	
Wavelength	1.54178 Å	
Crystal system	orthorhombic	
Space group	Pbca	
Z	32	
Unit cell dimensions	a = 20.103(2) Å	$\alpha = 90°$
	b = 34.492(2) Å	$\beta = 90°$
	c = 75.856(5) Å	$\gamma = 90°$
Volume	52597(7) Å^3	
Density (calculated)	1.07 g/cm^3	
Absorption coefficient	1.39 mm^{-1}	
Crystal shape	plate	
Crystal size	0.071 × 0.058 × 0.023 mm^3	
Crystal colour	red	
Theta range for data collection	2.3 to 30.0°	
Index ranges	$-13 \leq h \leq 12, -22 \leq k \leq 22, -49 \leq l \leq 22$	
Reflections collected	40728	
Independent reflections	7474 (R_{int} = 0.3034)	
Observed reflections	3845 (I > 2σ(I))	
Absorption correction	Semi-empirical from equivalents	
Max. and min. transmission	0.98 and 0.01	
Refinement method	Full-matrix least-squares on F^2	
Data/restraints/parameters	7474 / 13420 / 2673	
Goodness-of-fit on F^2	1.55	
Final R indices (I>2sigma(I))	R_1 = 0.104, wR_2 = 0.238	
Largest diff. peak and hole	0.85 and -0.28 eÅ^{-3}	

Table S2. Crystal structure, crystal data and structure refinement for **BT-3** (CCDC 2411869).

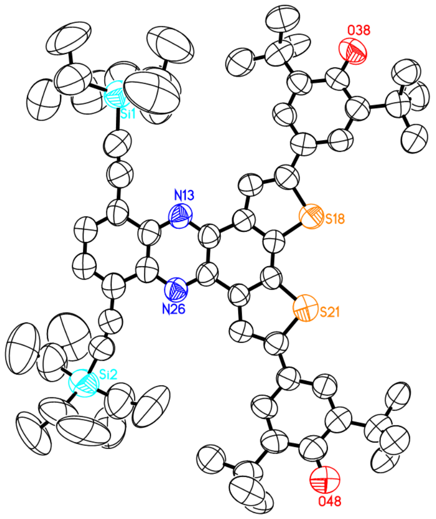

Empirical formula	$C_{66}H_{86}N_2O_2S_2Si_2$	
Formula weight	1059.66	
Temperature	200(2) K	
Wavelength	1.54178 Å	
Crystal system	orthorhombic	
Space group	Pbca	
Z	48	
Unit cell dimensions	a = 19.7031(8) Å	$\alpha = 90°$
	b = 34.7433(17) Å	$\beta = 90°$
	c = 114.993(6) Å	$\gamma = 90°$
Volume	78718(6) $Å^3$	
Density (calculated)	1.07 g/cm^3	
Absorption coefficient	1.39 mm^{-1}	
Crystal shape	prism	
Crystal size	0.110 × 0.042 × 0.032 mm^3	
Crystal colour	blue/bronze	
Theta range for data collection	2.4 to 45.0°	
Index ranges	$-18 \le h \le 11, -27 \le k \le 31, -101 \le l \le 104$	
Reflections collected	111630	
Independent reflections	29641 (R_{int} = 0.3339)	
Observed reflections	10249 ($I > 2\sigma(I)$)	
Absorption correction	Semi-empirical from equivalents	
Max. and min. transmission	0.96 and 0.68	
Refinement method	Full-matrix least-squares on F^2	
Data/restraints/parameters	29641 / 27282 / 3997	
Goodness-of-fit on F^2	1.43	
Final R indices (I>2sigma(I))	R_1 = 0.162, wR_2 = 0.303	
Largest diff. peak and hole	0.64 and -0.38 $eÅ^{-3}$	

Table S3. Crystal structure, crystal data and structure refinement for **BT-4** (CCDC 2411870).

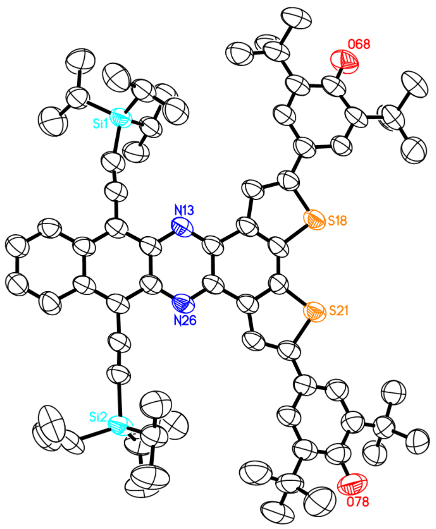

Empirical formula	$C_{71.50}H_{89.50}Cl_{4.50}N_2O_2S_2Si_2$	
Formula weight	1288.77	
Temperature	200(2) K	
Wavelength	1.54178 Å	
Crystal system	monoclinic	
Space group	$P2_1/c$	
Z	8	
Unit cell dimensions	a = 21.6902(14) Å	$\alpha = 90°$
	b = 11.8575(5) Å	$\beta = 97.512(5)°$
	c = 56.108(3) Å	$\gamma = 90°$
Volume	14306.7(14) $Å^3$	
Density (calculated)	1.20 g/cm^3	
Absorption coefficient	2.87 mm^{-1}	
Crystal shape	needle	
Crystal size	0.286 × 0.030 × 0.012 mm^3	
Crystal colour	green	
Theta range for data collection	3.8 to 46.1°	
Index ranges	$-19 \le h \le 20, -4 \le k \le 11, -52 \le l \le 51$	
Reflections collected	39835	
Independent reflections	11892 (R_{int} = 0.1712)	
Observed reflections	6734 ($I > 2\sigma(I)$)	
Absorption correction	Semi-empirical from equivalents	
Max. and min. transmission	1.00 and 0.25	
Refinement method	Full-matrix least-squares on F^2	
Data/restraints/parameters	11892 / 4813 / 1550	
Goodness-of-fit on F^2	1.08	
Final R indices (I>2sigma(I))	R_1 = 0.112, wR_2 = 0.247	
Largest diff. peak and hole	0.79 and -0.39 $eÅ^{-3}$	

6. Supplementary Spectroscopic Data

Figure S25 shows the linear absorption spectra of compound **BT-0** across the full measured linear spectral range (350-900 nm) with the corresponding σ_2. These spectra complement the energy-scale plots in the main text by providing an alternative visualization in wavelength units. Similarly, Figure S26 shows the full spectra in nanometers for compounds **BT-1** and **BT-2**, while **BT-3**, **BT-4**, and **BT-5** are shown in Figure S27.

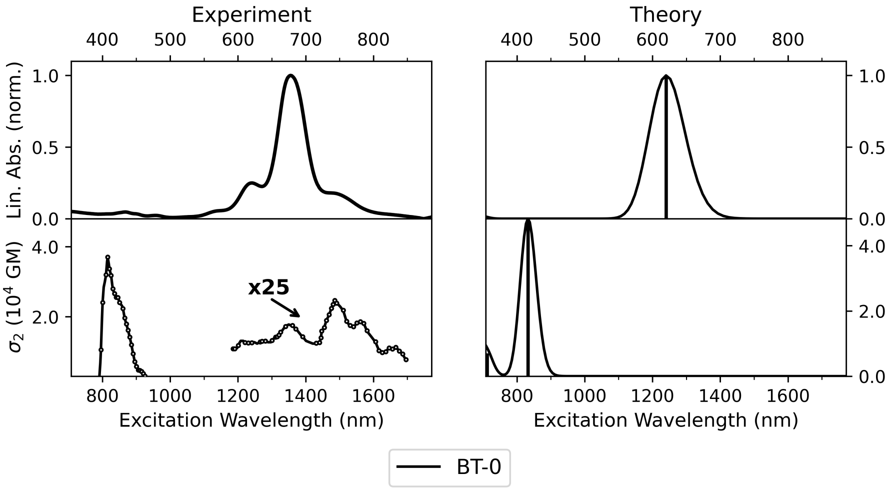

Figure S25. Comparison of the experimental 1PA and 2PA spectra of **BT-0** in benzene over the full measured range with the corresponding spectra calculated at the CAM-B3LYP-D3(BJ)/aug-cc-pVDZ PCM(C_6H_6) level of theory. The 25× magnification applies only to the low-energy band.

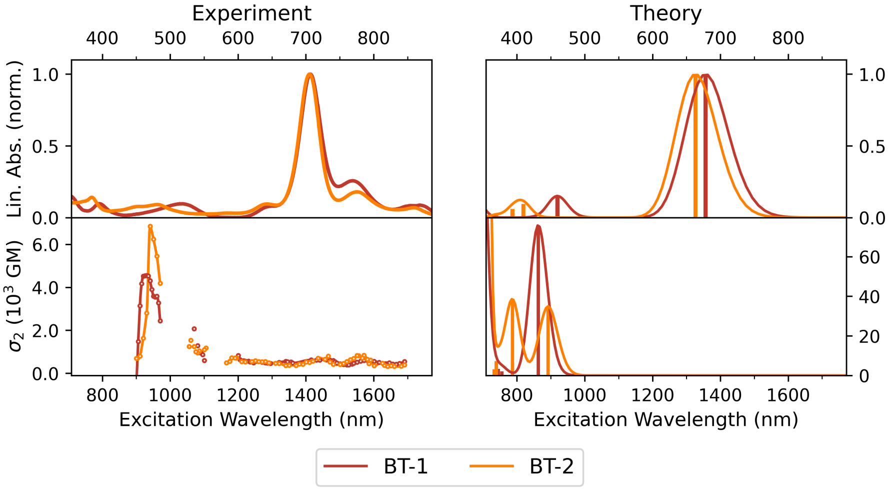

Figure S26. Comparison of the experimental 1PA and 2PA spectra of **BT-1** and **BT-2** in benzene over the full measured range with the corresponding spectra calculated at the CAM-B3LYP-D3(BJ)/aug-cc-pVDZ PCM(C_6H_6) level of theory.

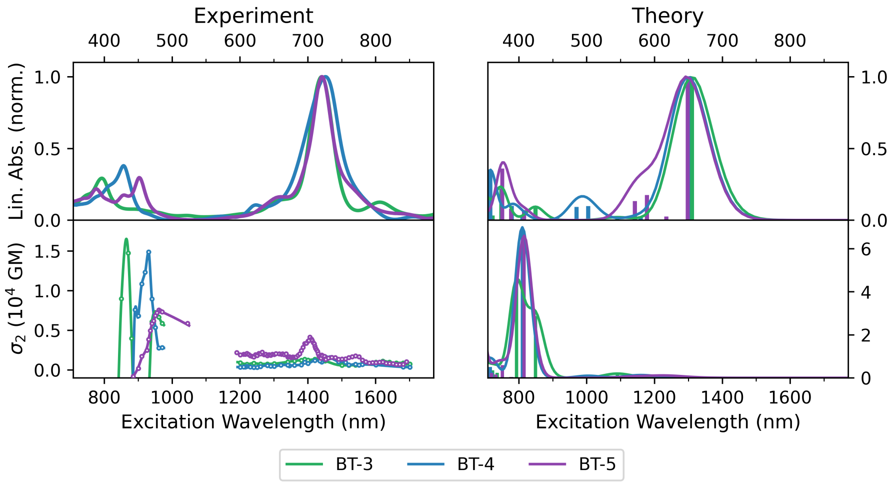

Figure S27. Comparison of the experimental 1PA and 2PA spectra of **BT-3**, **BT-4** and **BT-5** in benzene over the full measured range, with the corresponding spectra calculated at the CAM-B3LYP-D3(BJ)/aug-cc-pVDZ PCM(C_6H_6) level of theory.

7. Experimental Methods

7.1. Two-Photon Absorption Measurements

7.1.1. Sample Preparation

The two-photon absorption (2PA) measurements were conducted on solutions containing the derivatives of interest. These derivatives were dissolved in benzene to reach a concentration of 1.7 mM (for **BT-0**), 2 mM (for **BT-1**, **BT-2**) and 1 mM (for **BT-3**, **BT-4**, **BT-5**), all under a nitrogen atmosphere to avoid reduction reactions that might alter sample's properties. The prepared solutions were then placed into 2 mm path length Infrasil cuvettes (Starna 1/ST/C/I/2) to ensure accurate and consistent path length for the measurements.

7.1.2. Open-Aperture Z-Scan Technique

The 2PA properties of the solutions were assessed using the open-aperture Z-scan technique, a reliable method for determining nonlinear absorption characteristics.[11] A picosecond Nd:YAG laser (Ekspla PL2230) operating at a 50 Hz repetition rate was utilized, ensuring complete thermal relaxation between pulses. To cover a broad spectral range in the near-infrared region, the laser beam was modified using a harmonic generator and a parametric generator, achieving a narrow pulse width ($\lambda_p = 3.7$ nm). The laser pulses, characterized by a Gaussian temporal profile with a full width at half maximum (FWHM) of 18 ps, were spatially reshaped using a Gaussian filter. The transmittance T for each pulse and z-stage position was recorded by comparing the pulse energy after and before propagation through the sample, using a pyroelectric energy meter (Ophir Optronics PE9-C) for pulse energy measurements.

7.1.3. Analysis

The analysis of the open-aperture Z-scan traces focused on the absence of beam distortion, indicating that the transmittance changes were solely due to nonlinear absorption. For a temporally Gaussian pulse, the normalized transmitted energy as a function of sample position z is given by[12]:

$$T(z) = \frac{1}{\sqrt{\pi} q_0(z,0)} \int_{-\infty}^{\infty} \ln\left[1 + q_0(z,0)e^{-t^2}\right] dt, \tag{S1}$$

where $q_0(z,t) = \beta I_0 L_{\text{eff}}/(1+x^2)$, and $x = z/z_0$ with z_0 being the Rayleigh length, β the nonlinear absorption coefficient, I_0 the maximum irradiance, and L_{eff} the effective sample length. For $|q_0| < 1$, this equation can be simplified to a summation suitable for numerical evaluation:

$$T(z) = \sum_{m=0}^{\infty} \left[\frac{-q_0(z,0)}{(m+1)^{3/2}} \right]. \tag{S2}$$

Curve fitting of the Z-scan traces was performed using this equation. The 2PA cross section σ_2 was then calculated using the formula:

$$\sigma_2 = \frac{h\nu\beta}{N_A d}, \tag{S3}$$

where N_A is Avogadro's constant, $h\nu$ the photon energy, β the two-photon absorption coefficient, and d the concentration. The 2PA cross section is reported in Göppert–Mayer (GM) unit (1 GM = 10^{-50} cm^4 s molecule^{-1} photon^{-1}) and was evaluated for every measured excitation wavelength. Measurements at the same wavelength but different input peak irradiances have been performed to exclude multiphoton effects and to verify the nonlinear correction through the fitted beta.

7.1.4. Saturable Absorption

In addition to 2PA measurements, the influence of saturable absorption was also considered. Saturable absorbers can exhibit nonlinear transmission characteristics, where the absorption decreases with increasing light intensity, leading to effects such as reverse saturable absorption (RSA) at high intensities. This behavior was monitored to ensure accurate interpretation of the 2PA data and to distinguish between 2PA and saturable absorption effects.

8. Computational Methodology

8.1. Computational Details

All quantum chemical calculations were carried out using a development version of the Q-Chem 6.0 program package.[13] For geometry optimizations, frequency and excited-state calculations, the conductor-like polarizable continuum model (C-PCM) was used to simulate the solvation effects of benzene ($\epsilon = 2.3$).[14] The geometries of the investigated systems were optimized at the B3LYP-D3(BJ)/6-311G(d,p) level of theory. It should be noted that t-Bu and TIPS groups were replaced by Me and TMS groups, respectively. Frequency calculations at the same level of theory confirmed in each case that the stationary point is a local minimum. Using the range-separated hybrid exchange-correlation functional CAM-B3LYP[15] with Grimme's D3(BJ) dispersion correction[16,17] and aug-cc-pVDZ as the basis set,[18] the 100 lowest excited singlet states were computed within the Tamm-Dancoff approximation (TDA),[19] including the transition moments between excited states and the excited-state dipole moments. JMol[20] was used to visualize (transition) dipole moment vectors. In order to analyze the electronic structure of the most important states involved in the 2PA processes, attachment and detachment densities were also calculated. An isovalue of 0.002 was used to depict the densities.

8.2. Analysis

The UV-vis spectra were generated by convolving the computed stick spectra with Gaussian functions. The 2PA spectra were obtained from the calculated properties by applying a few-state model. For this purpose, the sum in Eq. (1) was formed over the first 100 excited singlet states. Since the denominator in Eq. (1) becomes larger with increasing energetic distance between the final state and the other excited states and the corresponding contributions thus become progressively smaller, this approximation is justified. For linearly polarized light with parallel polarization, the rotationally averaged 2PA strength (in atomic units) can be calculated from the obtained tensor as

$$\langle \delta^{2PA} \rangle = \frac{1}{15} \sum_{AB} (2S_{AB}S_{AB} + S_{AA}S_{BB}). \tag{S4}$$

According to Ref. 21, the macroscopic 2PA cross section (in cgs units), which is compared with the experiment, can then be determined as follows

$$\sigma_2 = \frac{4\pi^3 \alpha a_0^5 \omega^2}{c} \langle \delta^{2PA} \rangle g(2\omega, \omega_f, \Gamma), \tag{S5}$$

where α is the fine structure constant, a_0 the Bohr radius, ω the photon energy in atomic units, c the speed of light, and $g(2\omega, \omega_f, \Gamma)$ the lineshape function that describes spectral broadening effects. In our case, a Gaussian lineshape function

$$G(2\omega) = \frac{\sqrt{\ln 2}}{\Gamma\sqrt{\pi}} \exp\left[-\ln 2\left(\frac{2\omega - \omega_f}{\Gamma}\right)^2\right] \tag{S6}$$

was used again. Here, ω_f denotes the excitation energy of the final state f and Γ the half width at half maximum (HWHM). In accordance with the experiment, a HWHM ($= \frac{1}{2}\times$FWHM) of 0.1 eV was used throughout.

9. Experimentally determined 1PA and and 2PA properties of all investigated compounds

Tables S4 and S5 summarize the experimentally determined 1PA (Table S4) and 2PA (Table S5) properties of all investigated compounds.

Table S4. Absorption maximum of the one-photon excitation for the investigated systems in benzene. For all compounds, the maximum linear absorption is located in the low-energy band.

	Low-energy band		High-energy band	
	$E_a{}^a$ [eV]	$\lambda_a^{(1)}$ [nm]	$E_b{}^a$ [eV]	$\lambda_b^{(1)}$ [nm]
**BT-0**	1.83	678		
**BT-1**	1.75	708	2.39	519
**BT-2**	1.75	708	2.57	482
**BT-3**	1.72	721	3.12	397
**BT-4**	1.71	725	2.89	429
**BT-5**	1.72	725	2.57	482

[a] $E_{a,b}$ is the energy of the maximum of the peak in the absorption band.

Table S5. Measured 2PA parameters for the investigated systems in benzene. **BT-0** is listed for three different concentrations. **BT-3** has a double peak structure in the high-energy band.

	Low-energy band		High-energy band	
	$E_{max}{}^a$ [eV]	$\sigma_{2,max}$ [GM]	$E_{max}{}^a$ [eV]	$\sigma_{2,max}$ [GM]
**BT-0** (1.7 mM)	0.86	1010	1.52	35550
**BT-0** (0.85 mM)			1.51	50860
**BT-0** (0.35 mM)			1.52	51770
**BT-1**	0.79	600	1.34	4550
**BT-2**	0.93	900	1.32	6800
**BT-3**	0.77	1300	1.31	7200
			1.43	14800
**BT-4**	0.85	1650	1.30	14900
**BT-5**	0.88	4200	1.29	7550

[a] $E_{max} = \frac{hc}{\lambda_{in}}$ is the energy of the incident photons.

9.1. Concentration-Dependent 2PA Measurements

The concentration of the absorbing molecule significantly influences its nonlinear optical properties, particularly the interplay between saturable absorption (SA) and 2PA. As the concentration increases, the absorption process can begin to saturate, causing a deviation from the expected quadratic dependence of absorption on incident power. Consequently, the calculated GM values may not accurately reflect the true 2PA cross section of the material.

Concentration-dependent measurements provide crucial insights into the complex relationship between linear and nonlinear optical absorption processes. The onset of linear absorption occurs at approximately 1.4 eV. In more

concentrated solutions, SA initiates as a competing effect already at lower energies, potentially distorting the 2PA signal. In contrast, solutions with lower concentrations allow for 2PA measurements at higher energies before the competing SA becomes significant.
Additionally, one can observe, that the overall effect of SA is more pronounced in lower concentrated solutions due to the reduced number of molecules available for absorption. This leads to a more rapid onset of saturation and results in a steeper decline in nonlinear absorption at around 1.525 eV.
This results in a complex behavior of competing nonlinear effects within the BT solutions at certain photon energies, where the higher GM values observed at lower concentrations can be attributed to reduced saturation effects, allowing for a more accurate representation of the intrinsic 2PA cross section.

9.2. Reversed Saturable Absorption

Nonlinear behavior in the high intensity region can lead an additional non-linear absorption due to 2PA or 3PA. Also the transmittance can increase with laser intensity due to a saturation of the linear 1PA due to depletion of the ground state at high intensities. 1PA is occurring for **BT-0** starting at 1.5 eV. The high intensity laser pulse raises the ground state population to the first excited state. This excitation happens faster than its spontaneous relaxation and thus leads to the depletion of the ground state also called ground state bleaching.
Competing to this process there can be reverse saturable absorption (RSA) that will lead to an intensity dependent absorption. This can either be a sequential event (absorbing a photon from an excited state) or a multi-photon absorption where two or three photons are absorbed simultaneously.

The dependence of NLO response of the **BT-0** on the incident intensity in the high-energy regime has been investigated by adopting a wide range of input intensities and can be seen in Fig. S28. It can be clearly seen that there are overlaying effects, where in the low-intensity regime SA effects dominate. When the input peak intensity is increased, a valley in the normalized transmittance emerges at the focal point, indicating a typical response for reversed saturable absorption. In this case, the RSA is attributed to the large 2PA at this excitation energy. It is apparent that SA and nonlinear absorption coexist, with the shape of the trace as a result of competing nonlinear effects.

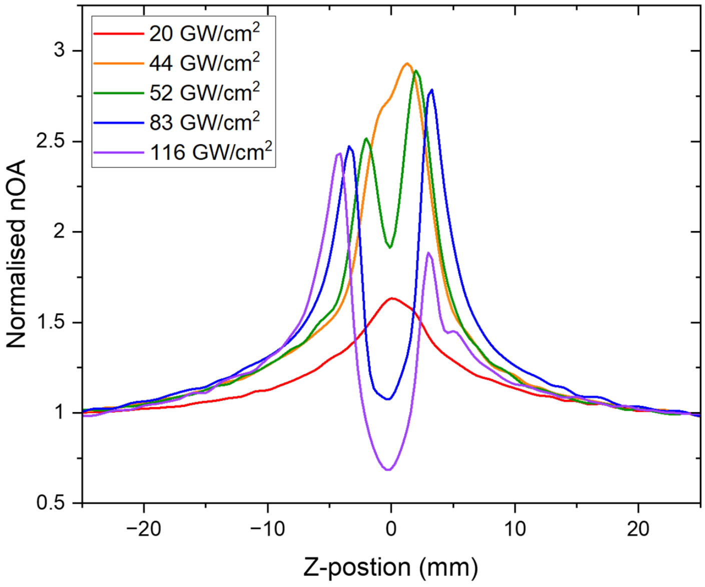

Figure S28. Z-san traces of **BT-0** with different input peak intensities at a fixed excitation energy (wavelength).

10. Calculated Excited-State Properties and Few-State Models

According to Alam *et al.*,[22] for Hermitian theories, the following generalized few-state model (GFSM) can be formulated for a 2PA process from the ground state into the final state f

$$\langle\delta^{2PA}_{\mathrm{GFSM}}\rangle = \sum_{i,j\neq 0} \frac{4|\vec{\mu}_{0i}||\vec{\mu}_{if}||\vec{\mu}_{0j}||\vec{\mu}_{jf}|}{15\Delta E_i \Delta E_j} \times \left(\cos\theta^{if}_{0i}\cos\theta^{jf}_{0j} + \cos\theta^{0j}_{0i}\cos\theta^{jf}_{if} + \cos\theta^{jf}_{0i}\cos\theta^{if}_{0j}\right), \tag{S7}$$

where the sum runs over selected excited states that determine the model. In the above expression, θ^{cd}_{ab} denotes the angle between the transition dipole moment vectors $\vec{\mu}_{ab}$ and $\vec{\mu}_{cd}$ and $\Delta E_a = \omega_a - \frac{\omega_f}{2}$. Eq. (S7) gives the same results as Eq. (1) (in combination with Eq. (S4)) if both include the same excited states, but can give a better insight into the corresponding 2PA process. In the following, for some 2PA processes, the contributions of the individual states are examined utilizing the GFSM.

BT-0

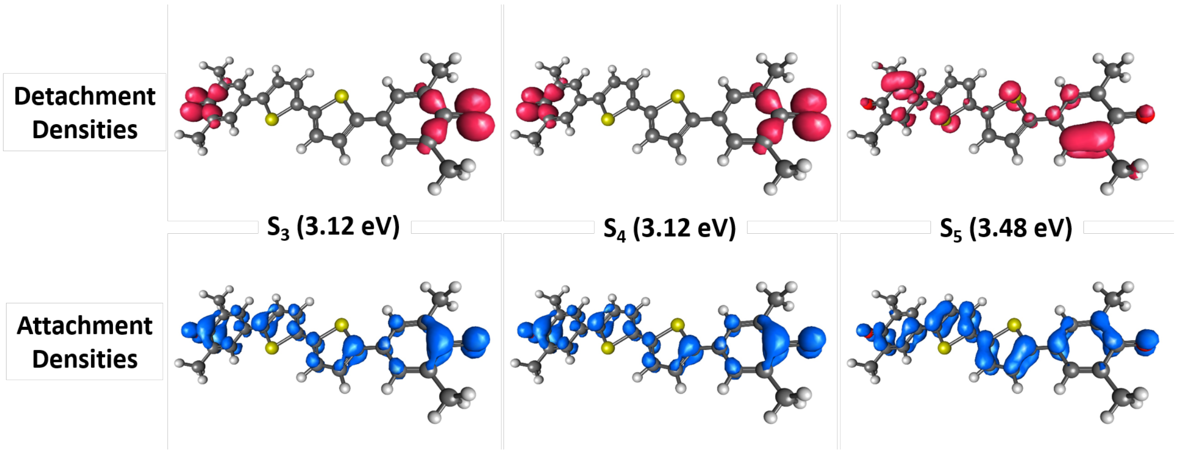

Figure S29. Attachment (blue) and detachment (red) densities of the other excited singlet states of **BT-0**, calculated at the CAM-B3LYP-D3(BJ)/aug-cc-pVDZ PCM(C_6H_6) level of theory. The corresponding densities of the first two excited singlet states are shown in Figure 5.

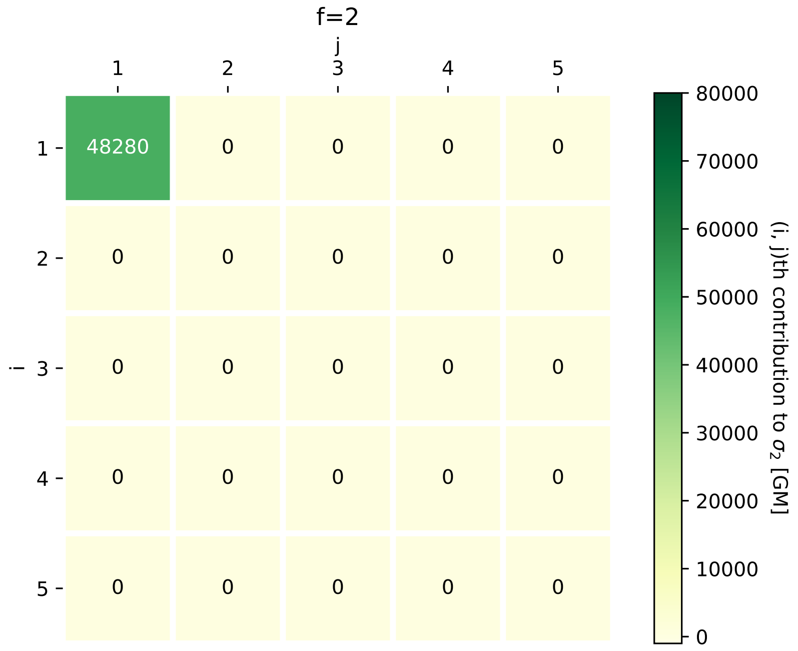

Figure S30. Contributions of the individual states to σ_2 using the GFSM for the 2PA process into the S_2 state of **BT-0**, calculated at the CAM-B3LYP-D3(BJ)/aug-cc-pVDZ PCM(C_6H_6) level of theory. Due to the centrosymmetry, the S_1 state makes the only contribution.

BT-1 and BT-2

Table S6. Excitation energies (ω_f), oscillator strengths, 2PA cross sections (σ_2), transition dipole moments from the ground state ($\langle 0|\vec{\mu}|f\rangle$), difference dipole moments ($\langle f|\vec{\overline{\mu}}|f\rangle$), and transition dipole moments from the first excited state to the final state ($\langle 1|\vec{\mu}|f\rangle$) for the first five and eight excited singlet states of **BT-1** and **BT-2**, respectively, calculated at the CAM-B3LYP-D3(BJ)/aug-cc-pVDZ PCM(C_6H_6) level of theory.

f	ω_f [eV]	osc. str. [a.u.]	σ_2 [GM]	$\lvert\langle 0\lvert\vec{\mu}\rvert f\rangle\rvert$ [a.u.]	$\lvert\langle f\lvert\vec{\overline{\mu}}\rvert f\rangle\rvert$ [a.u.]	$\lvert\langle 1\lvert\vec{\mu}\rvert f\rangle\rvert$ [a.u.]
**BT-1**						
1	1.83	3.519	41	8.862	1.069	-
2	2.70	0.528	114	2.826	1.295	0.522
3	2.87	0.018	75932	0.501	0.509	7.255
4	2.93	0.000	0	0.008	2.897	0.008
5	2.93	0.000	8	0.004	2.894	0.068
**BT-2**						
f	ω_f [eV]	osc. str. [a.u.]	σ_2 [GM]	$\lvert\langle 0\lvert\vec{\mu}\rvert f\rangle\rvert$ [a.u.]	$\lvert\langle f\lvert\vec{\overline{\mu}}\rvert f\rangle\rvert$ [a.u.]	$\lvert\langle 1\lvert\vec{\mu}\rvert f\rangle\rvert$ [a.u.]
1	1.87	3.474	45	8.709	1.123	-
2	2.78	0.003	34912	0.214	2.428	6.411
3	2.97	0.000	0	0.017	2.992	0.003
4	2.97	0.000	0	0.014	2.991	0.007
5	3.03	0.334	205	2.122	1.784	0.484
6	3.15	0.211	38011	1.651	2.982	3.631
7	3.35	0.063	7183	0.873	2.203	0.913
8	3.39	0.007	3144	0.283	1.054	0.958

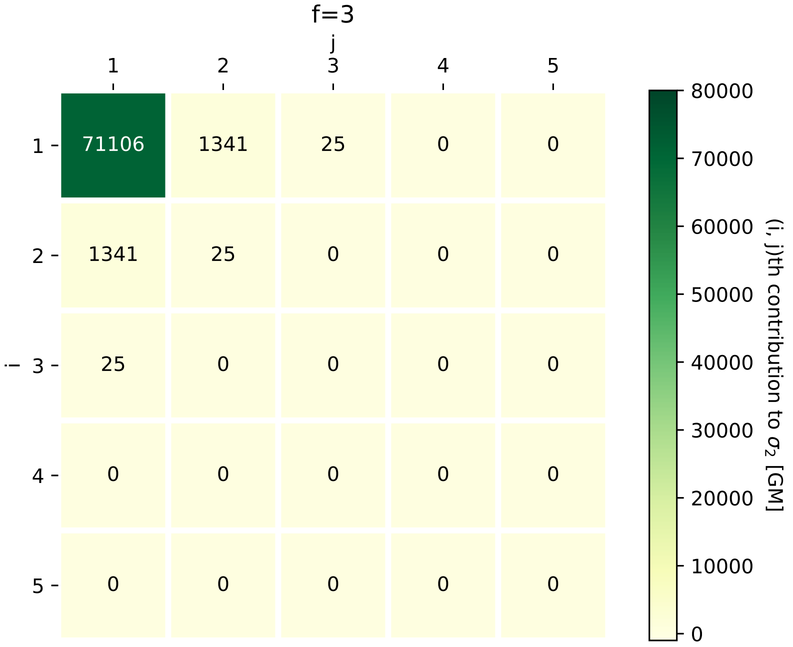

Figure S31. Contributions of the individual states to σ_2 using the GFSM for the 2PA process into the S_3 state of **BT-1**, calculated at the CAM-B3LYP-D3(BJ)/aug-cc-pVDZ PCM(C_6H_6) level of theory. The S_1 state still makes the only significant contribution. As the selection rules are somewhat relaxed due to the lower symmetry compared to **BT-0**, there are also contributions from other states, but these are negligible.

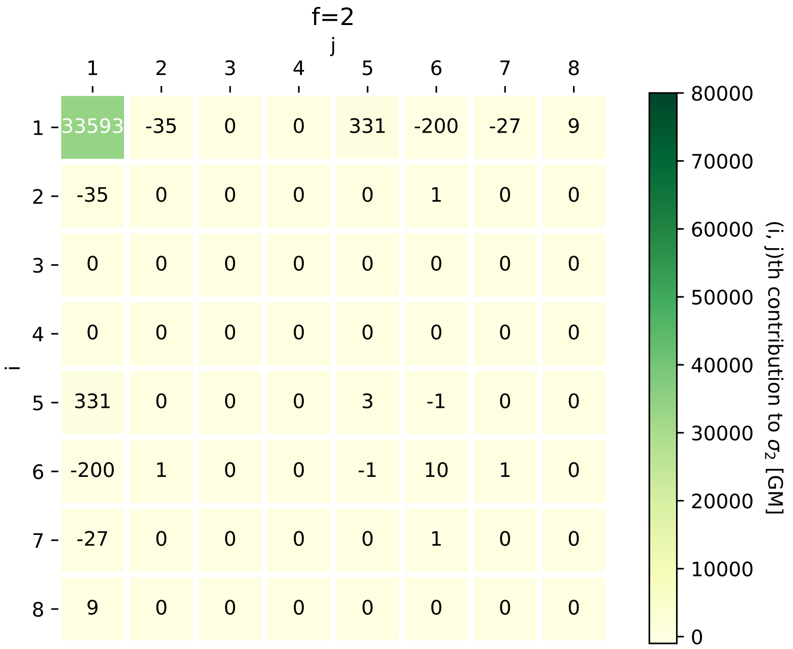

(a)

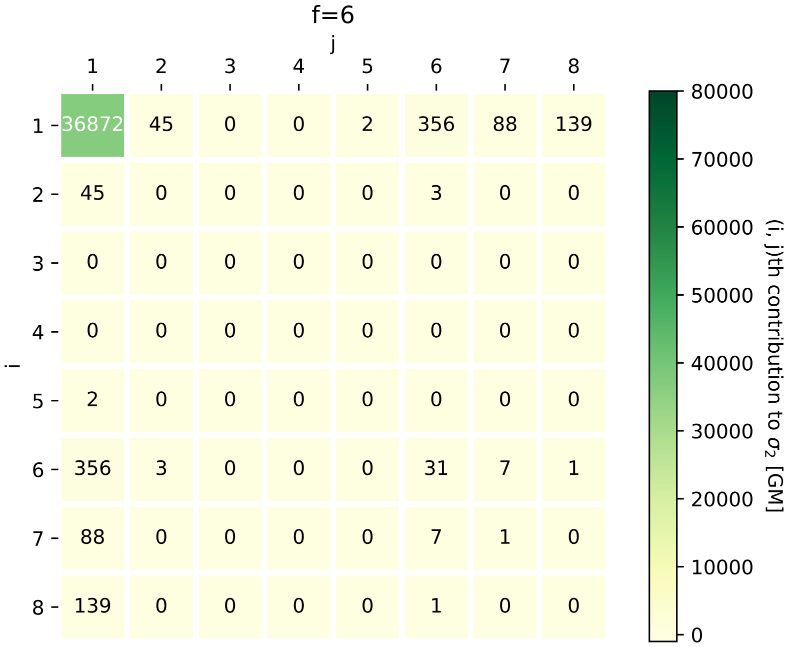

(b)

Figure S32. Contributions of the individual states to σ_2 using the GFSM for the 2PA process into the (a) S_2 and (b) S_6 states of **BT-2**, calculated at the CAM-B3LYP-D3(BJ)/aug-cc-pVDZ PCM(C_6H_6) level of theory. In both cases, the S_1 state still makes the only significant contribution. As the selection rules are somewhat relaxed due to the lower symmetry compared to **BT-0**, there are also contributions from other states, but these are negligible.

BT-3 to BT-5

Table S7. Excitation energies (ω_f), oscillator strengths, 2PA cross sections (σ_2), transition dipole moments from the ground state ($\langle 0|\vec{\mu}|f\rangle$), difference dipole moments ($\langle f|\vec{\overline{\mu}}|f\rangle$), and transition dipole moments from the first excited state to the final state ($\langle 1|\vec{\mu}|f\rangle$) for the first eight excited singlet states of **BT-3** to **BT-5**, calculated at the CAM-B3LYP-D3(BJ)/aug-cc-pVDZ PCM(C_6H_6) level of theory.

**BT-3**						
f	ω_f [eV]	osc. str. [a.u.]	σ_2 [GM]	$\lvert\langle 0\lvert\vec{\mu}\rvert f\rangle\rvert$ [a.u.]	$\lvert\langle f\lvert\vec{\overline{\mu}}\rvert f\rangle\rvert$ [a.u.]	$\lvert\langle 1\lvert\vec{\mu}\rvert f\rangle\rvert$ [a.u.]
1	1.89	3.236	39	8.355	1.085	-
2	2.27	0.067	1989	1.098	7.512	3.157
3	2.92	0.300	28650	2.047	3.833	5.188
4	2.99	0.000	0	0.004	3.772	0.005
5	2.99	0.000	0	0.004	3.773	0.010
6	3.05	0.001	0	0.133	2.922	0.002
7	3.10	0.011	202	0.379	2.182	0.537
8	3.13	0.007	44215	0.298	3.852	4.566
**BT-4**						
f	ω_f [eV]	osc. str. [a.u.]	σ_2 [GM]	$\lvert\langle 0\lvert\vec{\mu}\rvert f\rangle\rvert$ [a.u.]	$\lvert\langle f\lvert\vec{\overline{\mu}}\rvert f\rangle\rvert$ [a.u.]	$\lvert\langle 1\lvert\vec{\mu}\rvert f\rangle\rvert$ [a.u.]
1	1.91	3.172	43	8.235	1.099	-
2	2.14	0.084	1302	1.268	7.846	3.043
3	2.47	0.310	994	2.264	9.876	1.690
4	2.56	0.294	1	2.166	3.297	0.291
5	3.01	0.000	0	0.008	4.367	0.005
6	3.01	0.000	0	0.002	4.368	0.006
7	3.06	0.109	69725	1.203	0.743	6.797
8	3.18	0.314	376	2.006	2.907	0.565
**BT-5**						
f	ω_f [eV]	osc. str. [a.u.]	σ_2 [GM]	$\lvert\langle 0\lvert\vec{\mu}\rvert f\rangle\rvert$ [a.u.]	$\lvert\langle f\lvert\vec{\overline{\mu}}\rvert f\rangle\rvert$ [a.u.]	$\lvert\langle 1\lvert\vec{\mu}\rvert f\rangle\rvert$ [a.u.]
1	1.91	2.889	48	7.856	1.004	-
2	2.01	0.074	987	1.223	8.092	2.728
3	2.11	0.519	0	3.173	3.985	0.855
4	2.17	0.395	613	2.723	11.000	0.862
5	3.01	0.000	1	0.011	5.868	0.021
6	3.01	0.000	0	0.006	5.865	0.005
7	3.04	0.208	65812	1.673	0.881	6.865
8	3.19	0.234	376	1.729	3.315	0.540

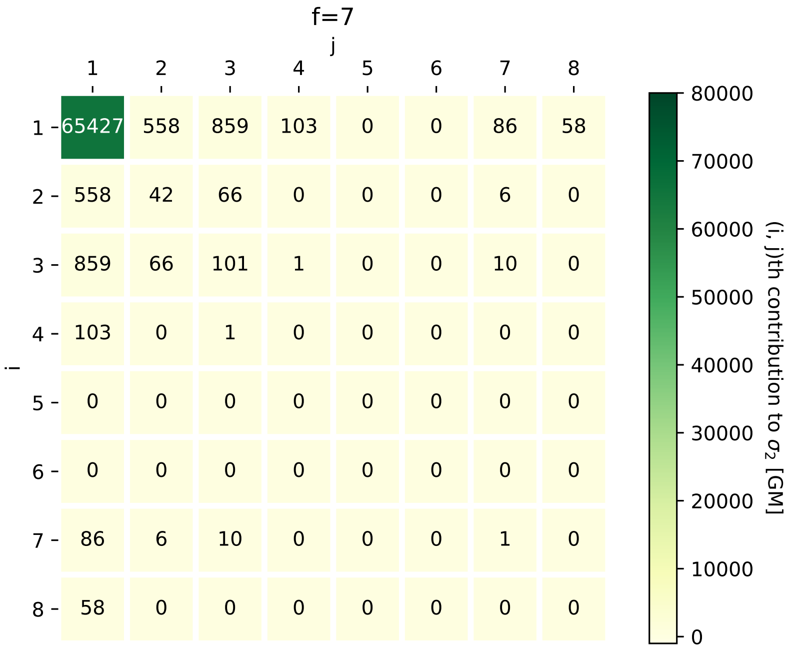

Figure S33. Contributions of the individual states to σ_2 using the GFSM for the 2PA process into the S_7 state of **BT-4**, calculated at the CAM-B3LYP-D3(BJ)/aug-cc-pVDZ PCM(C_6H_6) level of theory. The S_1 state still makes the only significant contribution. As the selection rules are somewhat relaxed due to the lower symmetry compared to **BT-0**, there are also contributions from other states, but these are negligible.

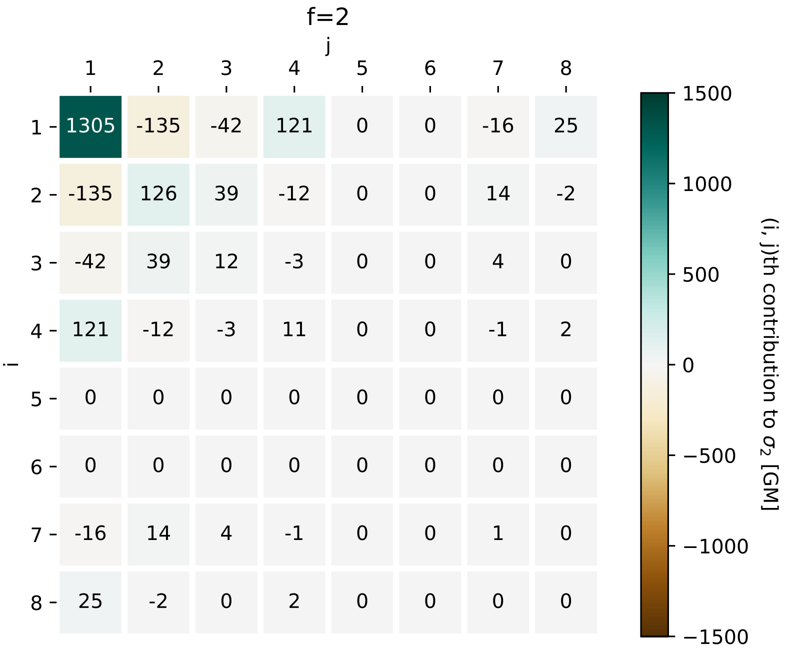

(a)

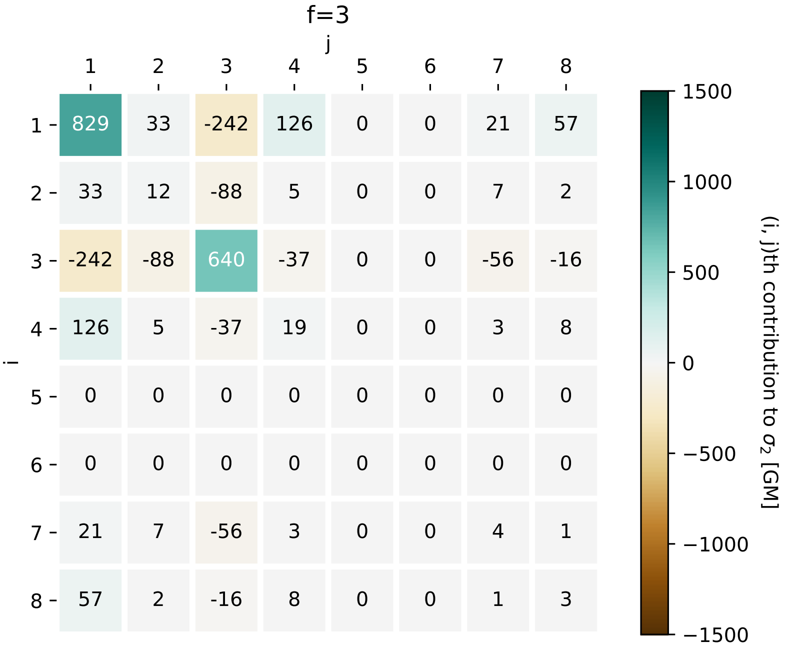

(b)

Figure S34. Contributions of the individual states to σ_2 using the GFSM for the 2PA process into the (a) S_2 and (b) S_3 state of **BT-4**, calculated at the CAM-B3LYP-D3(BJ)/aug-cc-pVDZ PCM(C_6H_6) level of theory. Since the respective final state has a non-zero oscillator strength and the dipole moment changes considerably upon excitation, the final state also makes a significant contribution in addition to the S_1 state. This is more pronounced for (b). However, due to the opposite orientation of the corresponding (transition) dipole moment vectors, the off-diagonal contributions are negative in both cases.

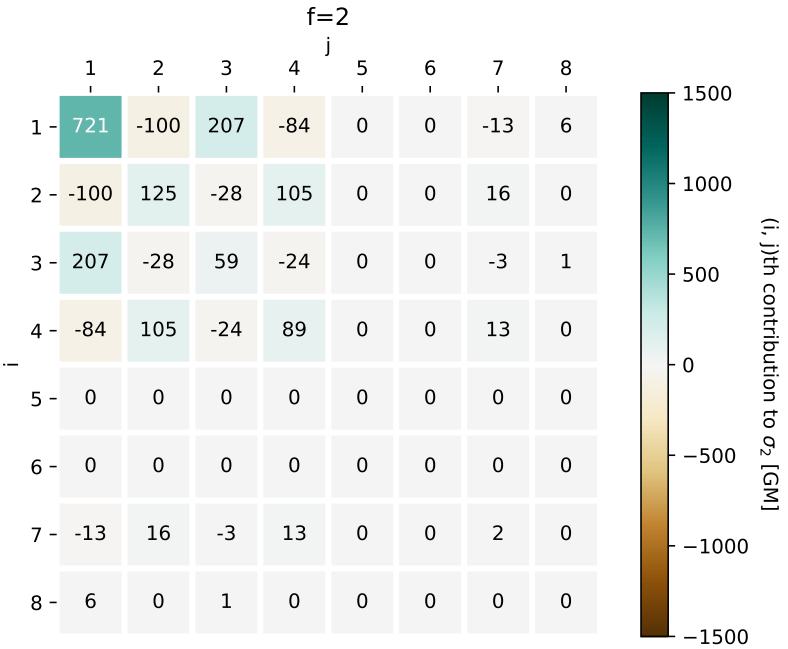

(a)

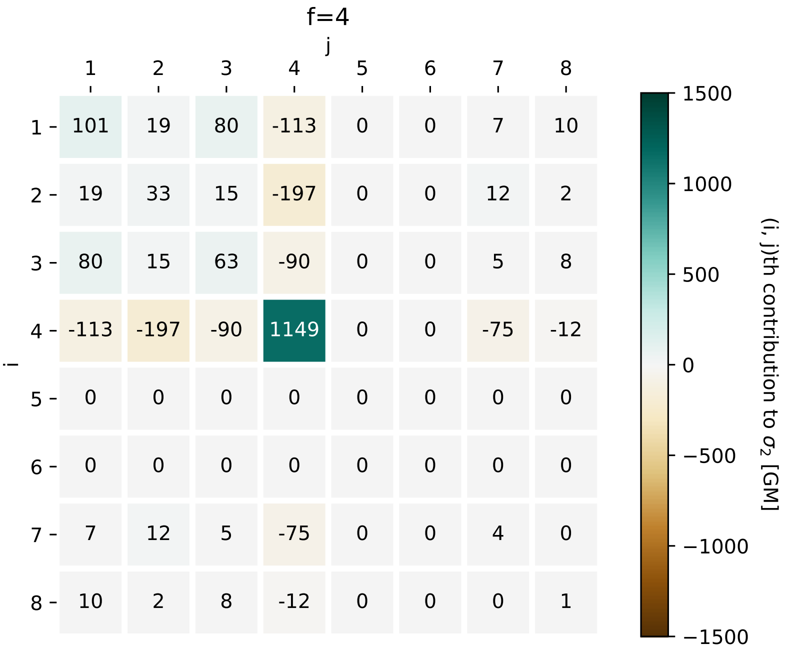

(b)

Figure S35. Contributions of the individual states to σ_2 using the GFSM for the 2PA process into the (a) S_2 and (b) S_4 state of **BT-5**, calculated at the CAM-B3LYP-D3(BJ)/aug-cc-pVDZ PCM(C_6H_6) level of theory. Since the respective final state has a non-zero oscillator strength and the dipole moment changes considerably upon excitation, the final state also makes a significant contribution in addition to the S_1 state. This is more pronounced for (b), also in comparison to the shorter acene in Figure S34b. However, due to the opposite orientation of the corresponding (transition) dipole moment vectors, the off-diagonal contributions are negative in both cases.

References

[1] G. R. Fulmer, A. J. M. Miller, N. H. Sherden, H. E. Gottlieb, A. Nudelman, B. M. Stoltz, J. E. Bercaw, K. I. Goldberg, *Organometallics* **2010**, *29*, 2176.
[2] L. Krause, R. Herbst-Irmer, G. M. Sheldrick, D. Stalke, *J. Appl. Crystallogr.* **2015**, *48*, 3.
[3] G. M. Sheldrick, *Acta Crystallogr. A* **2015**, *71*, 3.
[4] W. Harrer, H. Kurreck, J. Reusch, W. Gierke, *Tetrahedron* **1975**, *31*, 625.
[5] Y. Liu, P. M. Lahti, *Molecules* **2004**, *9*, 725.
[6] R. Rieger, D. Beckmann, A. Mavrinskiy, M. Kastler, K. Müllen, *Chem. Mater.* **2010**, *22*, 5314.
[7] Y. Xu, Y. Cui, H. Yao, T. Zhang, J. Zhang, L. Ma, J. Wang, Z. Wei, J. Hou, *Adv. Mater.* **2021**, *33*, e2101090.
[8] K. Fuchs, S. Medina Rivero, A. Weidlich, F. Rominger, N. Israel, A. A. Popov, A. Dreuw, J. Freudenberg, J. Casado, U. H. F. Bunz, *Angewandte Chemie (International ed. in English)* **2023**, *62*, e202305712.
[9] S. N. Intorp, M. Hodecker, M. Müller, O. Tverskoy, M. Rosenkranz, E. Dmitrieva, A. A. Popov, F. Rominger, J. Freudenberg, A. Dreuw, U. H. F. Bunz, *Angewandte Chemie (International ed. in English)* **2020**, *59*, 12396.
[10] Y. Satoh, C. Shi, *Synthesis* **1994**, *1994*, 1146.
[11] R. L. Sutherland, *Handbook of Nonlinear Optics*, volume 52 of *Optical Engineering. Series Editor*, Marcel Dekker, New York **1996**.
[12] M. Sheik-Bahae, A. A. Said, T.-H. Wei, D. J. Hagan, E. W. van Stryland, *IEEE J. Quantum Electron.* **1990**, *26*, 760.
[13] E. Epifanovsky, *et al.*, *J. Chem. Phys.* **2021**, *155*, 084801.
[14] J. Liu, W. Liang, *J. Chem. Phys.* **2013**, *138*, 024101.
[15] T. Yanai, D. P. Tew, N. C. Handy, *Chem. Phys. Lett.* **2004**, *393*, 51.
[16] S. Grimme, J. Antony, S. Ehrlich, H. Krieg, *J. Chem. Phys.* **2010**, *132*, 154104.
[17] S. Grimme, S. Ehrlich, L. Goerigk, *J. Comput. Chem.* **2011**, *32*, 1456.
[18] D. E. Woon, J. Dunning, Thom H., *J. Chem. Phys.* **1993**, *98*, 1358.
[19] S. Hirata, M. Head-Gordon, *Chem. Phys. Lett.* **1999**, *314*, 291.
[20] Jmol development team, Jmol: an open-source Java viewer for chemical structures in 3D.
[21] M. T. P. Beerepoot, D. H. Friese, N. H. List, J. Kongsted, K. Ruud, *Phys. Chem. Chem. Phys.* **2015**, *17*, 19306.
[22] M. M. Alam, M. Chattopadhyaya, S. Chakrabarti, *Phys. Chem. Chem. Phys.* **2012**, *14*, 1156.
